# Identification of fecal microbiome signatures associated with familial longevity and candidate metabolites for healthy aging

**DOI:** 10.1111/acel.13848

**Published:** 2023-05-02

**Authors:** Junli Gong, Sanxin Liu, Shisi Wang, Hengfang Ruan, Qianqian Mou, Ping Fan, Tao Chen, Wei Cai, Yongjun Lu, Zhengqi Lu

**Affiliations:** ^1^ Department of Colorectal Surgery, The Sixth Affiliated Hospital Sun Yat‐sen University Guangzhou Guangdong China; ^2^ Guangdong Provincial Key Laboratory of Colorectal and Pelvic Floor Diseases, The Sixth Affiliated Hospital Sun Yat‐sen University Guangzhou Guangdong China; ^3^ Guangdong Institute of Gastroenterology Guangzhou Guangdong China; ^4^ Key Laboratory of Human Microbiome and Elderly Chronic Diseases Ministry of Education Guangzhou Guangdong China; ^5^ Department of Neurology, Psychological and Neurological Diseases Research Centre The Third Affiliated Hospital of Sun Yat‐sen University Guangzhou Guangdong China; ^6^ Run Ze Laboratory for Gastrointestinal Microbiome Study School of Life Sciences of Sun Yat‐sen University Guangzhou Guangdong China; ^7^ South China Institute of Biomedicine Guangzhou Guangdong China

**Keywords:** familial longevity, fecal microbiome, healthy aging, pinane thromboxane A2

## Abstract

Gut microbiota associated with longevity plays an important role in the adaptation to damaging stimuli accumulated during the aging process. The mechanism by which the longevity‐associated microbiota protects the senescent host remains unclear, while the metabolites of the gut bacteria are of particular interest. Here, an integrated analysis of untargeted metabolomics and 16S rRNA gene sequencing was used to characterize the metabolite and microbiota profiles of long‐lived individuals (aged ≥90 years) in comparison to old‐elderly (aged 75–89 years), young‐elderly (aged 60–74 years), and young to middle‐aged (aged ≤59 years) individuals. This novel study constructed both metabolite and microbiota trajectories across aging in populations from Jiaoling county (the seventh longevity town of the world) in China. We found that the long‐lived group exhibited remarkably differential metabolomic signatures, highlighting the existence of metabolic heterogeneity with aging. Importantly, we also discovered that long‐lived individuals from the familial longevity cohort harbored a microbiome distinguished from that of the general population. Specifically, we identified that the levels of a candidate metabolite, pinane thromboxane A2 (PTA2), which is positively associated with aging, were consistently higher in individuals with familial longevity and their younger descendants than in those of the general population. Furtherly, functional analysis revealed that PTA2 potentiated the efficiency of microglial phagocytosis of β‐amyloid 40 and enhanced an anti‐inflammatory phenotype, indicating a protective role of PTA2 toward host health. Collectively, our results improve the understanding of the role of the gut microbiome in longevity and may facilitate the development of strategies for healthy aging.

## Graphical abstract

This study depicted the metabolite and microbiota trajectories across aging. Long‐lived subjects from the familial longevity cohort harbored a distinguished microbiome signature. A candidate metabolite, pinane thromboxane A2 (PTA2), was consistently higher in the individuals of familial longevity and their descendants. Functional analysis showed PTA2 enhanced an anti‐inflammatory phenotype, indicating a protective role of PTA2 toward host health. Our findings provide insights into the association between gut microbiome and longevity and may facilitate the development of strategies for healthy aging.

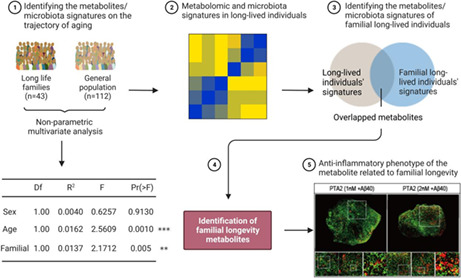

**Abbreviations**
AUC: area under curve
CSVD: cerebral small vessel disease
FDR: false discovery rate
G‐FL: grand elderly from the familial long‐living cohort
G‐GP: grand elderly from the general population
GPIIb/IIIa: glycoprotein IIb/IIIa
IBa1: ionized calcium binding adapter molecule 1
LDA: linear discriminant analysis
LEfSe: linear discriminant analysis effect size
O‐FL: old elderly from the familial long‐living cohort
O‐GP: old elderly from the general population
OSCs: organotypic slice cultures
PCoA: principal co‐ordinates analysis
PLS‐DA: partial least squares‐discriminant analysis
PTA2: pinane thromboxane A2
RDA: redundancy analysis
ROC: receiver operating characteristic
STAT: signal transducer and activator of transcription
STAT6: signal transducer and activator of transcription 6
TAMRA: tetramethylrhodamine
TBXA2R: thromboxane A2 receptor
TREM2: triggering receptor expressed on myeloid cells‐2
TXA2: thromboxane A2
UHPLC: ultra‐high performance liquid chromatography
UHPLC‐MS: ultra performance liquid chromatography‐tandem mass spectrometry
Y‐FL: young elderly from the familial long‐living cohort
Y‐GP: young elderly from the general population
βgal: β‐galactosidase

## 1 INTRODUCTION

During recent decades, the gut microbiota has been regarded as an active player in directing the health status of aging individuals by regulating digestive functions, neuronal activity, immunity, and resistance to pathogens infection (Franceschi et al., 2018; Rampelli et al., 2020; Wu et al., 2019). Investigations of the microbial data in older individuals often exhibited the existence of an “elderly‐type” microbiota configuration (Vaiserman et al., 2017), characterized by a loss of biodiversity, increase in the population of opportunistic pathobionts, and decrease in the population of probiotic bacteria (Cӑtoi et al., 2020). In contrast, studies of microbiomes associated with longevity have usually revealed an unexpectedly complex and extremely dynamic community, displayed by a higher bacterial diversity and accumulation of probiotic bacteria (Biagi et al., 2016; Kong et al., 2016). These findings indicate that the gut microbiota, rather than showing a mere consequence of aging, may play an active role in adaptation to the damaging stimuli accumulated during the aging process.

The fecal metabolome is a functional readout of bacterial activity and can be employed as an intermediate phenotype delivering host–bacterium interactions (Zierer et al., 2018). Aging manifests not only locally in the intestines (Jasper, 2020) but also systemically in various tissues and organs throughout the body (Kusumbe et al., 2014; Wyss‐Coray, 2016; Yousefzadeh et al., 2021). Therefore, to better elucidate the mechanisms by which the longevity‐associated microbiota protects the senescent host, metabolites produced by the gut bacteria that systemically circulate in the human body may be useful (Sato et al., 2021; Tuikhar et al., 2019). Generally, human longevity has intensive familial and genetic influences (Willcox et al., 2006). Studies have revealed that relatives of long‐lived individuals tend to have a significant survival advantage, a high possibility of being or becoming long‐lived, and a low risk of major age‐associated diseases such as cardiovascular and neurodegenerative diseases (O'Brien et al., 2007). Gut microbial metabolites have been demonstrated to be closely associated with neurochemical changes (Colombo et al., 2021; Li et al., 2019; Wu et al., 2021). Therefore, studies on the microbiota and metabolites of long‐lived people, especially familial long‐lived people, may help us discover candidate compounds that have protective effects on age‐associated diseases.

To this end, we conducted an integrated analysis of untargeted metabolomic and 16S rRNA gene sequencing to characterize and compare the metabolites and microbiota profiles of long‐lived (aged ≥90 years), old‐elderly (aged 75–89 years), young‐elderly (aged 60–74 years), and young to middle‐aged (aged ≤59 years) individuals. Besides, comparative analysis of the gut microbiota and metabolites was carried out between individuals from the familial longevity group and those from the general population. To our knowledge, this is the first study to construct both metabolite and microbiota trajectories across aging. Moreover, this is also the first attempt to characterize the microbiome features of individuals of the familial longevity. Altogether, our findings reveal the heterogeneity of gut microbiota and metabolites upon aging and in the long‐lived population. Meanwhile, we provide evidence that individuals with familial longevity have their own specific profiles in the distribution of gut microbiota and metabolites, which may have a protective effect on age‐associated diseases.

## 2 RESULTS

### 2.1 Sample collection and data analysis

A schematic overview of the study design is shown in Figure S1. Briefly, 155 participants, including 30 long‐living and 125 young age controls, were recruited from Jiaoling in Meizhou City (Guangdong province, China) between October 2019 and March 2020. Of the 30 long‐living individuals, 14 were recruited for familial longevity, and their descendants (*n* = 29) were recruited as part of the control group. With reference to the classification criteria of the World Health Organization, we grouped our cohort into four age groups: grand elderly (long‐lived, ≥90 years, group G), old elderly (75–89 years, group O), young elderly (60–74 years, group Y), and young to middle age (≤59 years, group M) (Table S1). First, the trajectories of the metabolomic and microbial profiles with aging in the cohort were characterized, followed by specific characterization of the metabolomic and microbiota profiles of the long‐lived individuals to identify the longevity‐associated biomarkers. Furthermore, we depicted the metabolomic and microbiota profiles of familial longevity since non‐parametric multivariate analysis of variance (ADONIS) revealed that not only age but also family were important contributing variables to the distribution of the microbial community. Finally, candidate metabolites that were supposed to promote longevity were explored, and their protective roles were evaluated through in vitro experiments.

### 2.2 Metabolomic profiling revealed the metabolite signatures on the trajectory of aging

**FIGURE 1:**
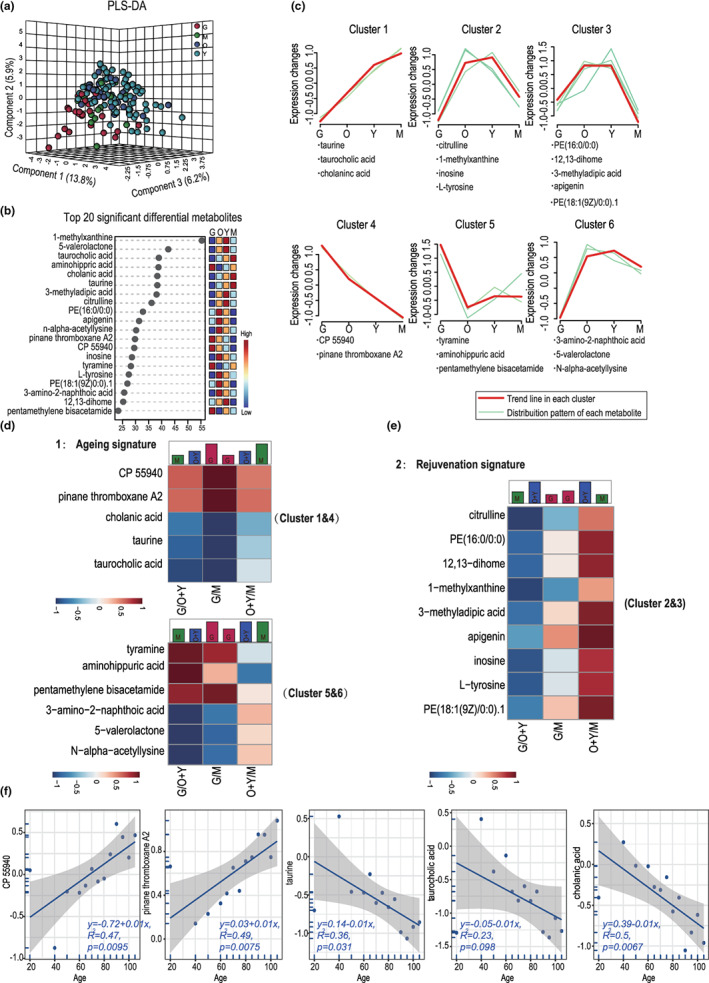
Metabolomic profiling revealed the metabolite signatures on the trajectory of aging. (a) Partial least squares‐discriminant analysis plot illustrating the distribution of fecal metabolites in long‐lived (grand elderly) (G, *n* = 30), old‐elderly (O, *n* = 25), young‐elderly (Y, *n* = 75), and middle‐aged and young (M, *n* = 15) individuals. (b) The top 20 most important metabolites in driving the separation among four groups. (c) The 20 most important metabolites were classified into 6 clusters according to their distribution trend upon aging. (d, e) Changes in the relative abundance of differentially distributed metabolites between G, O + Y, and M, according to the following signatures of abundance: 1. aging signature; 2. rejuvenation signature. Each signature is accompanied by models illustrating the relative abundance patterns of metabolites in the three groups (green, group M; blue, group O + Y; red, group G). (f) Line regression plot showing the positive or negative correlation between age and metabolites in Clusters 1 and 4.

To illustrate the fecal metabolite signatures of each age stage, a pattern recognition method with partial least squares‐discriminant analysis was employed to process the data from the ultra high performance liquid chromatography‐tandem mass spectrometry (UHPLC‐MS) based untargeted metabolomic detection. The long‐lived individuals (group G) had a systematically unique fecal metabolic profile, whereas the other three groups shared a relatively higher similarity (Figure 1a). Of the top 20 significant differential metabolites responsible for maximum separation (Figure 1b), 5 were enriched in group G, including aminohippuric acid, pentamethylene bisacetamide, pinane thromboxane A2 (PTA2), tyramine, and CP 55940, whereas 7 were enriched in group O, including PE(16:0/0:0) and PE(18:1(9Z)/0:0) belonging to phosphatidylethanolamine, apigenin belonging to the flavonoid subclass, and N‐alpha‐acetyllysine, inosine, l‐tyrosine, and 3‐amino‐2‐naphthoic acid, which are involved in amino acid and nucleotide metabolism. In addition, 1‐methylxanthine, 5‐valerolactone, 3‐methyladipic acid, citrulline, and 12,13‐dihome were enriched in group Y, whereas taurocholic acid, taurine, and cholanic acid, which are related to bile acid and taurine metabolism, were highly enriched in group M (Figure 1b). According to their distribution pattern on the aging profile, the 20 metabolites were classified into 6 clusters (Figure 1c). In combination with this classification (Table S2), we re‐grouped the 20 metabolites into an aging signature (Clusters 1 and 4, Clusters 5 and 6) or rejuvenation signature (Clusters 2 and 3) (Figure 1d,e). The abundance of PTA2 and CP 55940 in Cluster 4 increased with age, while the abundance of taurine, taurocholic acid, and cholanic acid in Cluster 1 decreased with age. Through linear regression analysis, we demonstrated that metabolites belonging to Clusters 1 and 4 were significantly correlated with age (Figure 1f). Metabolites listed in Clusters 2 and 3 showed a rejuvenation signature, with a relatively lower detection in both groups M and G, but a relatively higher detection in individuals from groups O and Y. Clusters 5 and 6 listed the metabolites with increased or decreased levels in group G, indicating that in addition to the metabolic changes due to aging, the long‐lived group exhibited a specific metabolomic signature. Taken together, these results demonstrated the existence of metabolic heterogeneity during aging. Notably, the long‐lived group exhibited remarkably different metabolomic signatures.

### 2.3 Aging‐related trajectories of gut microbiota

**FIGURE 2:**
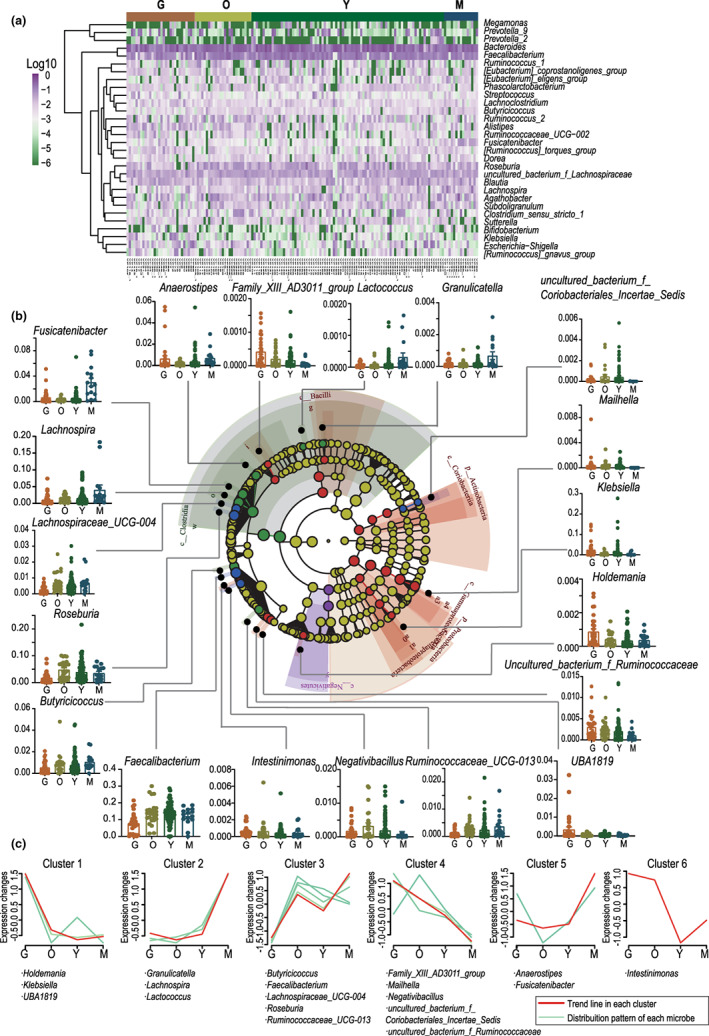
Aging‐related trajectories of gut microbiota. (a) Heatmap showing the top 30 abundant genera distributed in each sample (G, brown; O, light green; Y, dark green; M, blue). (b) Supervised analysis of 16S rRNA gene sequencing data with linear discriminant analysis (LDA) effect size comparing the microbiota at the genus level in groups G, O, Y, and M (LDA ≥2). (c) Classification of the 19 differentially distributed genera according to their distribution trend over the aging process.

Aging has been proposed to be characterized by an increasing abundance of subdominant species, and trajectories of aging‐related gut microbiota have previously been depicted in an Italian cohort (Biagi et al., 2016). To explore whether conservative changes occurred in the gut microbiota upon aging, the distribution of the microbiota at each age stage was characterized in our cohort. An overview of the distribution of microbiota across all age stages is illustrated in Figure 2a, which shows that *Bacteroide*s and *Faecalibacterium* were highly abundant across all age stages, whereas *Megamonas* and *Prevotella_*2 was rarely detected. Using principal coordinate analysis (PCoA) based on weighted UniFrac distances of the genus relative abundance matrix and permutational multivariate analysis of variance, we confirmed the discrimination of the gut microbiota among the groups (*p* = 0.011) (Figure S2A). The distinctiveness of the gut microbiota in different age stages was also evaluated through supervised redundancy analysis (RDA), which showed that age was a significant contributing factor to the differentiation of the fecal microbiota (Figure S2B). Linear discriminant analysis (LDA) effect size (LEfSe) was employed to identify the representative genera at each age stage; the results revealed 19 differentially abundant genera with an LDA score higher than 2.0 (Figure 2b). These differentially distributed bacteria mainly belonged to five classes, including Bacilli, Clostridia, Negativicutes, Gammaproteobacteria, and Coriobacteria. Butyrate producers belonging to Clostridia (*Fusicatenibacter*, *Lachnospira*, *Lactococcus*, *Anaerostipes*, *Butyricicoccus*, *Ruminococcaceae_UCG_013*, *Granulicatella*, *Faecalibacterium*, *Roseburia*, *Negativibacillus*, *Lachnospiraceae_UCG_004*), were mostly enriched in groups M and O, and their abundance cumulatively decreased with aging (Figure 2b).

Based on their trajectory upon aging, the 19 differential taxa were categorized into 6 clusters (Table S3). As shown in Figure 2c, Cluster 4 consisted of taxa whose abundance increased with age, while Cluster 3, which consisted of taxa associated with butyrate production, decreased with age. The latter has been reported in other cohorts of longevity, regardless of dietary habits or lifestyles. Cluster 1 included G‐specific taxa whose abundance was significantly enriched in group G but was similar among the control groups; Cluster 2 included the taxa whose abundance was only enriched in people aged under 60 years (group M); Cluster 5 showed a less distinct signature with Cluster 2, which included taxa enriched in M, but a less depleted pattern in the elders; Cluster 6 included only one taxon, *Intestinimonas*, whose abundance was higher in groups G and O, considerably depleted in group Y, and less depleted in group M. Collectively, these results described the existence of aging‐associated gut microbiota and depicted the age‐related trajectories of gut microbiota.

### 2.4 A multi‐omics meta‐analysis of metabolites and microbes in individuals from all age stages

On the basis of the profiling of the gut metabolites and microbes in individuals from each age stage, a multi‐omics meta‐analysis was used to investigate the interactions between metabolites and microbial genus (*r* = 0.4, *p* < 0.05) (Figure S3). The result showed that several metabolites including CP55940, pinane thromboxane A2, 5‐valerolactone and microbes including members from the Ruminococcaceae family, *Intestinimonas*, *Alistipes*, *Lactococcus* and Eubacterium_coprostanoligenes_group occupied important positions in the interaction network and interacted closely with each other, which might be defined as core metabolites or core bacteria. Of them, CP55940 and pinane thromboxane A2, being positively correlated with aging, showed positive interactions with most of the core bacteria. While, 5‐valerolactone, being identified to have a particularly low distribution level in the group G, showed negative interactions with most of the core bacteria. Beside of these, the associations between the majority of the bacteria or metabolites were much more scattered, indicating that they might play an accompanying role rather than a key role during the age‐related processes.

### 2.5 Characterization of the specific metabolomic and microbiota signatures in long‐lived individuals

**FIGURE 3:**
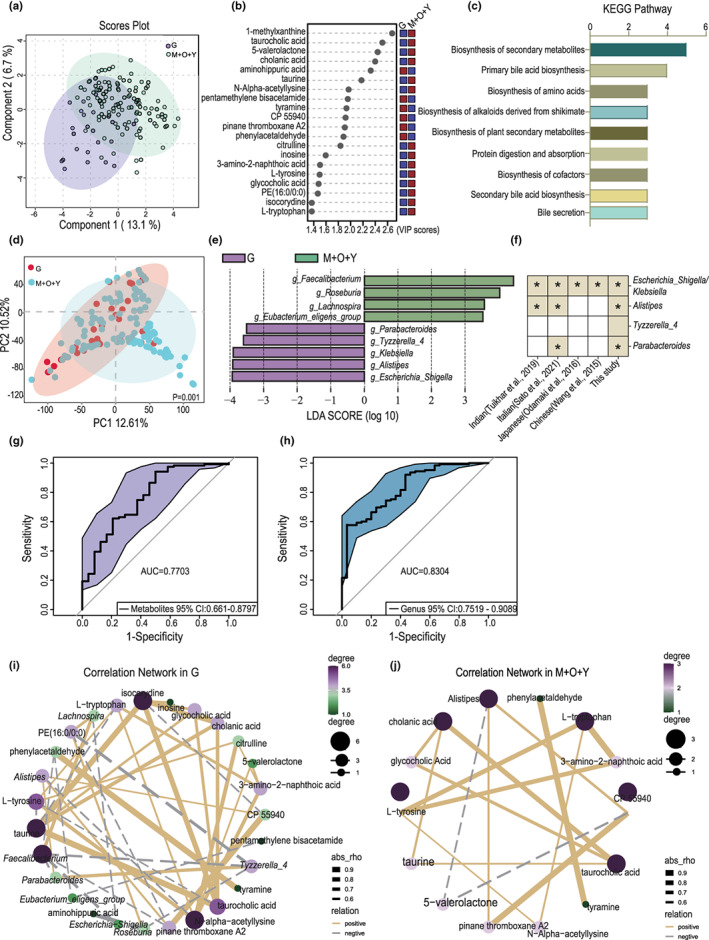
Distinct metabolomic and microbiota profiles existed in long‐living individuals. (a) Partial least squares‐discriminant analysis of the fecal metabolome of long‐living individuals (G, *n* = 30) and their young‐age controls (M + O + Y, *n* = 125). (b) Variable influence on projection plot showing the top 20 most important metabolites in driving the separation of the two groups. (c) Involved KEGG pathway (consisting of ≥3 metabolites in each pathway) of the differentially distributed metabolites. (d) Principal coordinate analysis plots based on the Unweighted UniFrac distance between gut microbiota in group G and group M + O + Y (*p* = 0.001). (e) Supervised analysis of 16S rRNA gene sequencing data with linear discriminant analysis (LDA) effect size (LDA ≥3.5). (f) Integrated analysis of longevity‐enriched bacteria across different studies. The asterisk (*) illustrates the shared genera in not less than two studies. (g, h) Receiver operating characteristic curves used to evaluate the metabolites and bacterial candidates differentiating group G from the control group. (i, j) Correlation network providing an overview of the relations between metabolites and bacterial candidates in groups G and M + O + Y, respectively.

As demonstrated above, long‐lived individuals have specific metabolomic and microbiota signatures. To better elucidate the fecal biomarkers of long‐lived individuals, we singled out the long‐lived population (group G) for comparison with other young‐aged populations (groups O + Y + M). For metabolomic features, the preliminary selection of characteristic metabolites was completed using the VIP value to identify the significant metabolites responsible for maximum separation in the PLS‐DA score plot (Figure 3a). The features of the top 20 metabolites are shown in Figure 3b. Among them, 1‐methyxanthine, taurocholic acid, 5‐valerolactone, cholanic acid, taurine, N‐alpha‐acetyllysine, citrulline, inosine, 3‐amino‐2‐naphthoic acid, l‐tyrosine, glycocholic acid, PE(16:0/0:0), isocorydine, and l‐tryptophan were enriched in group M + O + Y, while aminohippuric acid, pentamethylene bisacetamide, tyramine, CP 55940, PTA2, and phenylacetaldehyde were enriched in group G. These metabolites were involved in various KEGG pathways, including the biosynthesis of secondary metabolites, primary bile acid biosynthesis, and amino acid biosynthesis (Figure 3c).

As for microbiota features, the PCoA disclosed a significant difference in the microbiota taxonomic profiles between group G and the control group (*p* = 0.001) (Figure 3d). Using LEfSe (LDA ≥ 3.5) analysis, we revealed a significant enrichment of *Parabacteroides*, *Tyzzerella_4*, *Klebsiella*, *Alistipes*, and *Escherichia_Shigella*, as well as a depletion of *Faecalibacterium*, *Roseburia*, *Lachnospira*, and Eubacterium eligens_group in the long‐lived group (Figure 3e). Among them, *Escherichia/Shigella/Klebsiella*, *Alistipes*, and *Parabacteroides* have been reported previously in long‐living cohorts recruited from India, Italy, Japan, and China (Biagi et al., 2016; Kong et al., 2019; Odamaki et al., 2016; Tuikhar et al., 2019; Wang et al., 2015) (Figure 3f). The calculated area under the receiver operating characteristic (ROC) curve (AUC) of differentially distributed fecal metabolites and bacteria exhibited values of 0.7703 and 0.8304, respectively (Figure 3g,h), indicating that they are useful biomarkers for long‐term prediction. Consistent with previously proposed opinions that rearrangement in the gut microbiota co‐occurrence network occurs in long‐lived individuals, we observed a differential illustrated correlation network in group G compared with the control group (Figure 3i,j). Collectively, these results demonstrate that long‐living individuals have remarkably differentiated metabolomic and microbiota characteristics. Notably, a conserved microbiota exists in this long‐lived population.

### 2.6 Familial long‐lived individuals harbored distinct metabolomic and microbiota signatures

**FIGURE 4:**
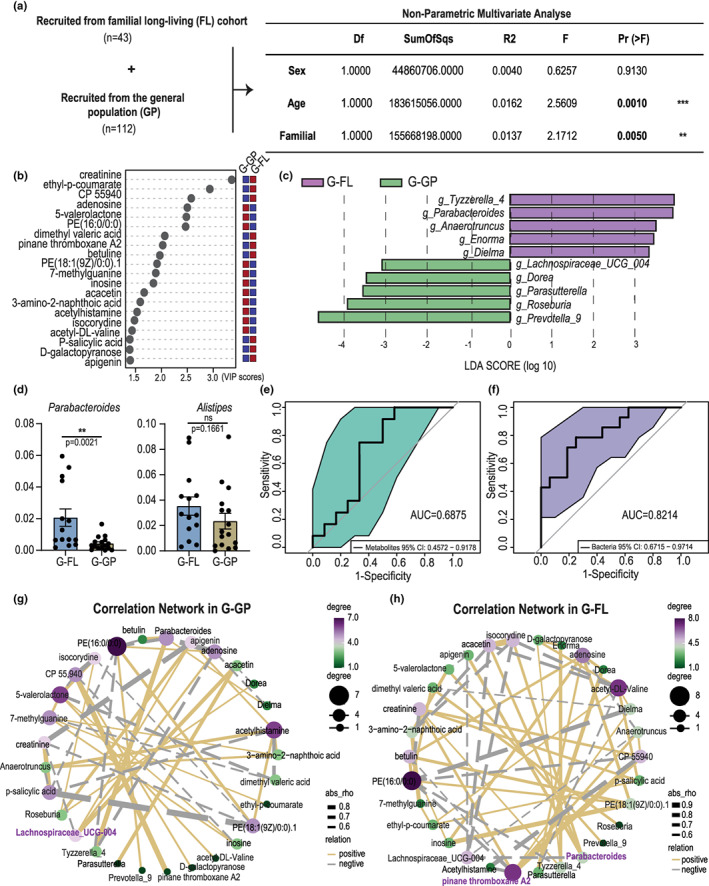
Familial long‐living individuals harbor distinct metabolomic and microbiota signatures. (a) Non‐parametric multivariate analysis showing family as a significant important variable in the distribution of fecal microbiota; (b) The variable influence on projection plot showing the top 20 most important metabolites driving the separation between familial long‐living individuals (G‐FL, *n* = 14) and long‐living individuals from the general population (G‐GP, *n* = 16). (c) Supervised analysis of 16S rRNA gene sequencing data with linear discriminant analysis effect size comparing the microbiota between groups G‐FL and G‐GP (LDA ≥3.5) (d). Boxplots showing the distribution of *Parabacteroides* and *Alistipes* between groups G‐FL and G‐GP. (e, f) Receiver operating characteristic curves used to evaluate the metabolites and bacterial candidates differentiating group G‐FL from group G‐GP. (g, h) Correlation network showing an overview of the relationship between metabolites and bacteria in groups G‐FL and G‐GP, respectively. ***p < 0.001; **p < 0.01. Wilcoxon rank‐sum text. ns, not significant. Each dot represents an individual.

Generally, human longevity has strong familial and genetic influences. As shown in Table S1, 14 long‐lived individuals in our cohort were recruited from familial longevity (G‐FL), in contrast to 16 long‐lived individuals recruited from the general population (G‐GP). Importantly, through non‐parametric multivariate analysis, we identified here that family served as an important contributing factor to the distribution of fecal microbiota (Figure 4a). Therefore, a comparative analysis was performed between G‐FL and G‐GP to elucidate the gut metabolomic and microbiota features of familial long‐lived individuals. To this end, we first characterized the top 20 significant metabolites responsible for maximum separation (Figure 4b). We identified an enrichment of nine metabolites, including creatinine, ethyl‐p‐coumarate, CP 55940, dimethyl valeric acid, PTA2, betuline, p‐salicylic acid, d‐galactopyranose, and apigenin in group G‐FL, and adenosine, 5‐valerolactone, and the other nine metabolites exhibited more abundant in group G‐GP compared to those of the G‐FL.

Next, the distribution of the microbiota from G‐FL and G‐GP was also explored. The results showed that the individuals from G‐FL harbored a distinct microbiota signature compared to that from the G‐GP, as indicated by the LEfSe algorithms (Figure 4c). In total, 10 predominant bacterial genera were screened, including *Tyzzerella*_4, *Parabacteroides*, *Anaerotruncus*, *Enorma*, and *Dielma*, which were enriched in group G‐FL, and *Prevotella*_9, *Roseburi*a, *Parasutterell*a, *Dorea*, and *Lachnospiraceae_UCG_004*, which were enriched in group G‐GP (Figure 4c). Previously, *Parabacteroides* and *Alistipes* were identified as two longevity‐enriched genera. Here, we showed that only *Parabacteroides* was highly abundant in familial long‐living individuals (Figure 4d). ROC curves generated from the differential metabolites and bacteria showed AUC values of 0.6875 and 0.8214, respectively, illustrating good performance in differentiating G‐FL from G‐GP (Figure 4e,f). Consistently, a completely different correlation network was revealed for groups G‐GP and G‐FL, with CP 55940 and Lachnospiraceae_UCG_004 being the two most important nodes in G‐GP, whereas PTA2 and *Parabacteroides* were the two most important nodes in the network in G‐FL (Figure 4g,h). Additionally, considering that differential genetic background or other undefined environmental factors might exist in our recruited population, we analyzed the microbiota and metabolic data of individuals in G‐FL. We found that subgroups existed in this group (Figure S4), and that eating habits as well as lifestyle (Table S4) only contributed partly to the structure of microbiota in G‐FL (*p* > 0.1, mantel_test). Thus, we suggested that the distribution of differential microbiome in G‐FL might be attributed to genetic background. Overall, these results revealed that although there were subgroups in G‐FL, a distinct metabolomic and microbiota signatures still could be observed between them and the long‐lived individuals from the general population.

### 2.7 PTA2 was more enriched in the familial long‐lived population and positively correlated with age

**FIGURE 5:**
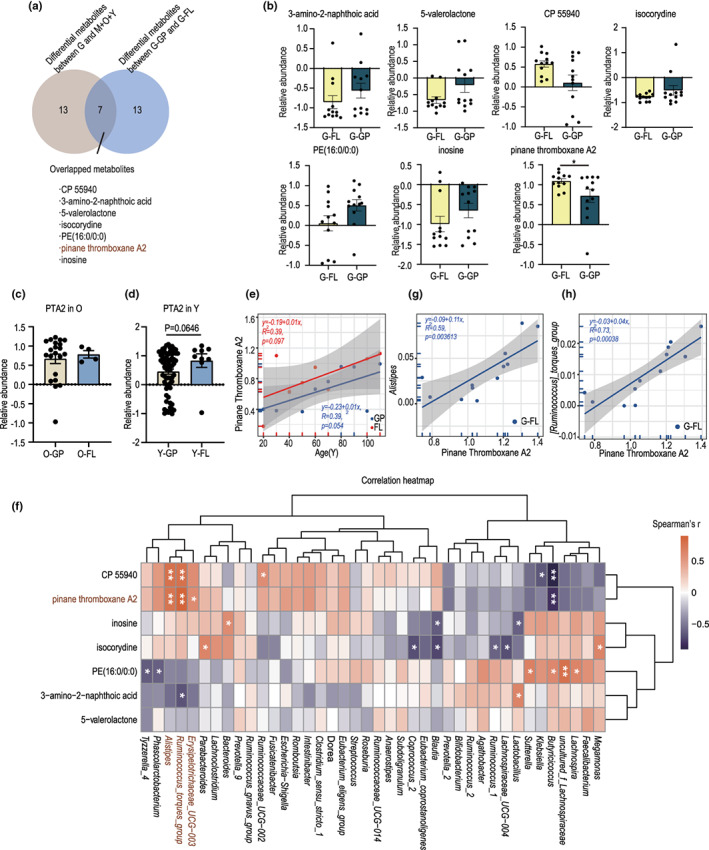
PTA2 was more enriched in the familial long‐living individuals and positively correlated with age. (a) Venn diagram showing the overlapped metabolites between differential distributed metabolites in groups G and G‐FL; (b) Boxplots showing the relative abundance of the seven overlapped metabolites. *p* Value between the groups G‐FLand G‐GP was calculated using two‐tailed Wilcoxon rank‐sum tests (False discovery rate (FDR) < 0.1). (c) Boxplots showing the distribution of PTA2 between old elderly from the familial long‐living cohort (O‐FL) and the general population (O‐GP); (d) Boxplots showing the distribution of PTA2 between young elderly from the familial long‐living cohort (Y‐FL) and the general population (Y‐GP); group Y‐FL tended to have a higher abundance of PTA2 (*p* = 0.0646). P values between groups G‐FL and G‐GP, O‐FL and O‐GP, and Y‐FL and Y‐GP were calculated using two‐tailed Wilcoxon rank‐sum tests. (e) Line regression plot showing that PTA2 is positively correlated with age in the whole population; (f) Heatmap showing the correlation between the seven overlapped metabolites and the top 40 abundant genera in group G‐FL. (g, h) Line regression plot showing that PTA2 is significantly positively correlated with the distribution of *Alistipes* and *Ruminococcus_torques_group*. **p < 0.01; *p < 0.05. Wilcoxon rank‐sum text. ns, not significant.

Familial longevity is considered a representative group in which genetics plays an important role in determining the lifespan. Nevertheless, our current analysis showed that the overall microbiota and metabolomic features of familial longevity were significantly different from those of long‐lived individuals in the general population (Figure 4). We then questioned whether the familial longevity associated microbiome jointly participated in influencing familial longevity. The metabolome has been regarded as a readout of fecal microbiota; hence, we intended to start our research from the perspective of metabolites. First, we screened for metabolites using a Venn diagram to illustrate those differentially distributed in both groups G and G‐FL. In total, seven metabolites were screened: CP 55940, 3‐amino‐2‐naphthoic acid, 5‐valerolactone, isocorydine, PE (16:0/0:0), PTA2, and inosine (Figure 5a) (Table S5). Only PTA2 showed a significantly higher abundance in group G‐FL according to the Mann–Whitney test (*p* = 0.045) (Figure 5b). In addition to group G‐FL, group Y‐FL but not O‐FL tended to have a higher abundance of PTA2 than its control group (Y‐GP) (*p* = 0.0646) (Figure 5c,d). Moreover, across all four age groups, PTA2 was considerably more abundant in familial longevity members than in the general population (Figure 5e). Spearman's correlation analysis showed that PTA2 in group G‐FL was significantly positively correlated with the relative abundance of *Alistipes*, *Ruminococcus*_*torques_group*, and *Erysipelotrichaceae_UCG‐003* in group G (Figure 5f) (Table S6), which is consistent with the observations in the interaction network between metabolites and microbes in individuals from all age stages (Figure S3); Besides, this result was further verified using linear regression analysis (Figure 5g,h). Collectively, these results confirmed that PTA2 was more enriched in familial long‐living individuals than in the general population, but whether it has a protective role towards healthy aging needs to be demonstrated.

### 2.8 PTA2 potentiates Aβ40‐specific phagocytic efficiency of microglia and enhances the anti‐inflammatory phenotype

PTA2 is a stable analog of thromboxane A2 (TXA2), and the binding of TXA2 to its cognate receptor thromboxane A2 receptor (TBXA2R) can increase the expression of glycoprotein IIb/IIIa (GPIIb/IIIa) on platelet membranes, leading to platelet activation and aggregation (Byrne et al., 1997). In the brain, the inability of microglia to clear misfolded proteins is associated with neurodegeneration, which may lead to a high prevalence of age‐associated cognitive decline (Minhas et al., 2021). Since the main source of TXA2 in the central nervous system is microglia (Giulian et al., 1996), we intended to explore whether PTA2 plays an anti‐inflammatory role in microglia, including enhancement of the Aβ‐specific phagocytic efficiency of microglia and blocking the increase of TXA2 during the process of phagocytosis of Aβ40 by microglia.

**FIGURE 6:**
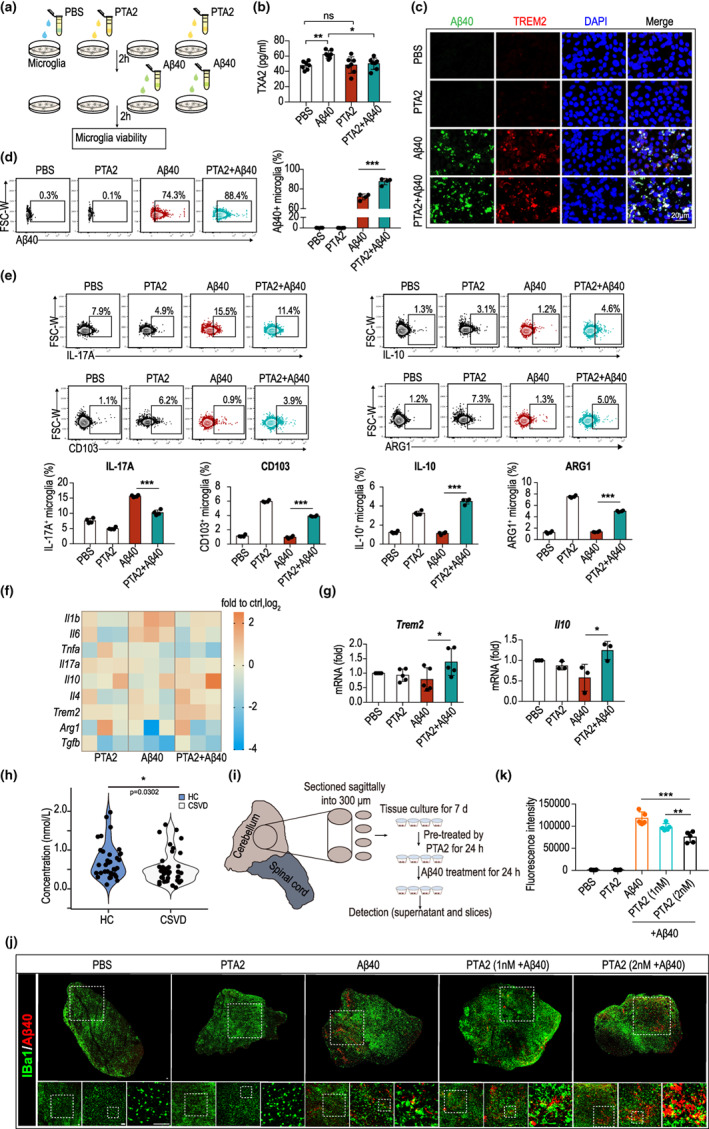
PTA2 potentiates Aβ40‐specific phagocytic efficiency of microglia and enhances the anti‐inflammatory phenotype by activating STAT6 signaling. (a) Experimental design. Mouse microglia were pretreated with PTA2 for 2 hr and then fed Aβ40 to observe the Aβ40 clearance efficiency and production of inflammatory factors 2 hr later. (b) Concentration of TXA2 in the supernatant of the microglial culture medium assessed with enzyme‐linked immunosorbent assay *p < 0.05, **p < 0.01, using one‐way ANOVA (mean ± standard deviation). (c) Representative images of double staining of Aβ40 (green) and the phagocytosis receptor TREM2 (red). Blue shows DAPI staining of nuclei. Scale bar: 20 μm. (d) Phagocytic efficiency of microglia was assessed with flow cytometric analysis. Experiments were repeated three times. (e) Expression of the pro‐inflammatory factor IL‐17A and M2 markers IL‐10, ARG1, and CD103 was assessed with flow cytometry. Experiments were repeated three times. (f) Heatmap showing the mRNA levels of inflammatory factors in each group evaluated using real‐time PCR. (g) Quantification showed that microglia after PTA2 pretreatment and stimulation with Aβ40 upregulated the expression of the phagocytosis‐associated gene TREM2 and the anti‐inflammatory gene IL‐10. (h) The distribution of serum PTA2 in healthy people and patients with CSVD. (i) Experimental design. Wild‐type organotypic slice cultures (OSCs) were pretreated with PTA2 (1 nM or 2 nM) for 24 h. A β40 peptides (25 mg/mL) labeled with carboxytetramethylrhodamine (TAMRA) were added in OSCs to activate phagocytosis of microglia. The supernatant of culture medium as well as the culture slices were subjected for further analysis. (j) Representative images of confocal microscopy showed that TAMRA‐Amyloid‐β (1–40) peptides (red) were phagocytized in IBa1^+^ microglia (green) as revealed by co‐localization. (k) Residual TAMRA fluorescence intensity of Aβ40 in the medium of OSCs was assessed with Agilent BioTek Synergy multimode microplate reader. CSVD: cerebral small‐vessel disease, **p < 0.01, ***p < 0.001; by one‐way ANOVA (mean ± standard deviation). Scale bar = 50 μm. Experiments were repeated for 3 times.

According to previous reported studies, PTA2 at 10 μM has the largest effect in inhibiting the aggregation of human platelets (Nicolaou et al., 1979). Therefore, to test our hypothesis, mouse microglia were pretreated with 10 μM PTA2 for 2 h and then fed with Aβ40 to activate microglial phagocytosis. The Aβ40 clearance efficiency and the production of inflammatory factors were observed 2 h later (Figure 6a). As expected, we observed that the intensity of TXA2 increased after Aβ40‐mediated stimulation of microglia, which was blocked by PTA2 treatment (Figure 6b). Furthermore, double immunofluorescence staining showed that the phagocytosis receptors TREM2 (red) and Aβ40 (green) were highly co‐localized, confirming enhanced Aβ40 phagocytosis in PTA2‐pretreated microglia compared to those treated with Aβ40 alone (Figure 6c). This result is consistent with that of our flow cytometric analysis (Figure 6d), which revealed that pretreatment with PTA2 reduced the number of IL‐17A^+^ pro‐inflammatory microglia and elevated the number of IL‐10^+^, ARG1^+^, and CD103^+^anti‐inflammatory phagocytes 2 h after Aβ40 phagocytosis (Figure 6e). The mRNA expression levels of various inflammatory mediators were also assessed (Figure 6f). We found that PTA2 pretreatment dramatically elevated the expression of the anti‐inflammatory gene *IL10* and phagocytosis‐associated gene *TREM2* (Figure 6g). We also observed a mild decrease in the expression levels of the pro‐inflammatory genes *IL1B*, *IL6*, and *TNFA* and a slight increase in the expression of the anti‐inflammatory genes *IL4* and *TGFB*, although the difference was not statistically significant (Figure 6f).

Next, we investigated whether PTA2, at physiological concentrations, could provide protective effects. Firstly, we analyzed the distribution of serum PTA2 levels in healthy individuals using LC–MS/MS, while patients with cerebral small vessel disease (CSVD) served as non‐healthy controls. Our results revealed that the concentration of PTA2 in healthy individuals ranged from 0.109 to 1.986 nM, which was significantly higher than that in CSVD patients (0.028 to 1.710 nM) (Figure 6h). Since PTA2 acts in a multi‐cell environment in vivo, we used organotypic slice cultures instead of microglial cells to investigate its phagocytosis‐promoting effect. We pretreated wild‐type organotypic slice cultures (OSCs) with PTA2 (1 nM or 2 nM) for 24 h and then added carboxytetramethylrhodamine‐labeled (TAMRA) Aβ40 peptides (25 mg/mL) to stimulate phagocytosis in microglia. After 24 h, we collected the supernatant of the culture medium and fixed the slices (Figure 6i). Our results indicated that more microglia were activated after TAMRA‐Aβ40 (1–40) stimulation, resulting in an enlarged cell body. Double immunofluorescent staining of IBa1 (green) and TAMRA‐Aβ40 (1–40) (red) confirmed enhanced Aβ40 phagocytosis in PTA2‐pretreated organotypic slice cultures, particularly at the higher concentration (2 nM), compared to Aβ40‐treated alone or lower PTA2 concentration (1 nM) (Figure 6j). The significantly reduced residual TAMRA fluorescence intensity in the supernatant of the culture medium further validated the results (Figure 6k). Taken together, these findings suggest that PTA2 pretreatment adjusted the inflammatory milieu in microglia after Aβ40 phagocytosis, promoting the phagocytosis and clearance of Aβ40 by microglia.

### 2.9 The protective role of PTA2 is associated with the activation of STAT6 signaling in microglia

**FIGURE 7:**
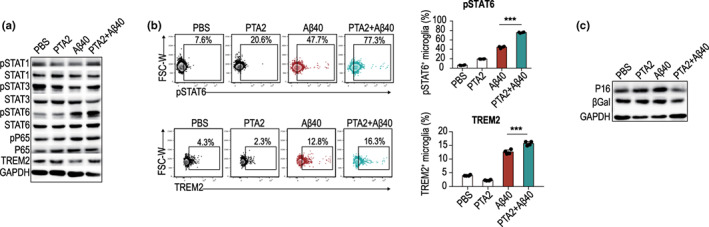
PTA2 activates STAT6 signaling to upregulate the expression of TREM2 in microglia. (a) Activation of TREM2 and other M1/M2‐relevant transcriptional factors was analyzed with western blotting. (b) Flow cytometric analysis of pSTAT6 and TREM2 in microglia 2 h after stimulation with Aβ40 and PTA2 pretreatment for 2 h. (c) Western blot analysis of the protein expression of β‐galactosidase and P16 in microglia with stimulation of Aβ40 after PTA2 pretreatment. Experiments were repeated three times. *p < 0.05, ***p < 0.001, using one‐way ANOVA (mean ± standard deviation).

STAT family members are important to the cellular inflammatory environment. Our previous study found that STAT6 signaling is essential for microglia, owing to their phagocytic function (Cai et al., 2019). Therefore, we explored a series of pathways to explain the molecular mechanism by which PTA2 enhances Aβ40 clearance by microglia. However, Aβ40‐activated microglia after PTA2 pretreatment peculiarly increased TREM2 expression by activating other M1/M2‐relevant transcriptional factors, including STAT6, as analyzed through western blotting (Figure 7a). Flow cytometry analysis indicated that STAT6 signaling was indeed activated and the expression of the phagocytic receptor TREM2 was upregulated (Figure 7b). Senescent cells often have increased lysosomal β‐galactosidase (βgal) activity, which is responsible for senescence‐associated βgal expression. P16 plays an important role in the initiation and maintenance of cellular senescence (Meng et al., 2003; Wang et al., 2006). Using western blotting experiments, we confirmed the interaction between PTA2 and the aging‐related proteins βgal and P16, which were suppressed by stimulation with Aβ40 and PTA2 pretreatment (Figure 7c). Therefore, we suggested that the protective role of PTA2 is associated with the activation of STAT6 signaling, which upregulated of the expression of TREM2 in microglia to prevent cellular senescence.

## 3 DISCUSSION

In this study, we characterized the metabolite and microbiota profiles of long‐lived individuals and constructed both metabolite and microbiota trajectories across aging. We noted that discrimination of microbiota and metabolites existed in long‐living individuals, as well as in individuals among different age stages. That is, there are age‐specific responses in terms of microbiome. Importantly, we observed that long‐lived individuals from the familial longevity cohort harbored a microbiome distinct from that of the general population. Specifically, we identified a candidate metabolite, PTA2, which was positively associated with aging and was present in consistently high levels in individuals with familial longevity and their younger descendants. Functional analysis revealed that PTA2 potentiates Aβ‐specific phagocytic efficiency of microglia and enhances the anti‐inflammatory phenotype.

To the best of our knowledge, this study presents the first systematic analysis of metabolic profiles with aging. Previously, the fecal metabolic feature of longevity was characterized by Tuikhar et al. (2019), who revealed that β‐alanine, gamma‐aminobutyric acid, and DL‐2‐aminoisobutyric acid were differentially distributed in centenarians (Tuikhar et al., 2019). In addition, Sato et al. (2021) investigated the gut microbiome and bile acid profiles in centenarians and their younger controls and revealed that unique secondary bile acids generated from centenarian‐enriched bacteria had a protective role against type II diabetes (Sato et al., 2021). Here, we identified 20 metabolites that were differentially distributed between the long‐living and control groups. Of these, a decrease in taurocholic, cholanic, and glycocholic acids, which are related to the metabolism of taurine, was observed in the long‐living individuals. These metabolites have been suggested as candidate biomarkers for anti‐aging, healthy lifespan, and rejuvenation (Heubi et al., 2015; Warden et al., 2020; Wójcik et al., 2010). In this study, however, they showed an opposite trend with increasing age and were even depleted in the long‐living animals, indicating that they did not confer longevity. Interestingly, we observed that two additional metabolites (PTA2 and CP 55940) were positively correlated with age. PTA2 is a carbocyclic analog of the highly unstable TXA2, acting as a TBXA2R antagonist that inhibits coronary artery contraction and platelet aggregation (Byrne et al., 1997). CP 55940 is a potent cannabinoid receptor agonist that has been explored for its efficacy in perturbing AD and other neurodegenerative states (Soto‐Mercado et al., 2021). Thus, our study demonstrated that the accumulation of beneficial compounds with aging may play a protective role in healthy aging.

The gut microbiota in the long‐living population has been well characterized (Biagi et al., 2016; Cӑtoi et al., 2020; Kong et al., 2016; Sato et al., 2021), and trajectories of aging‐related gut microbiota have previously been depicted in an Italian cohort (Biagi et al., 2016). To explore the conservative changes in microbiota upon aging, we characterized the distribution of microbiota at each age stage in our cohort. Consistent with a previous report that microbial ecosystems in long‐lived individuals harbored fewer butyrate producers and more Proteobacteria than other individuals, we also identified a cumulative decrease in the abundance of butyrate producers with aging. In addition, enrichment of *Klebsiella*, one of the members of Proteobacteria, was observed in long‐lived individuals. Furthermore, we observed an increase in the relative abundance of UBA1819 and *Intestinimonas* with aging. UBA 1819 and *Intestinimonas* are phylogenetically closely related to *Faecalibacterium* and have been reported to produce butyrate. Thus, these results indicate that the intestinal milieu at one specific age stage might limit the expansion of one type of butyrate producer and promote another. The shift in butyrate producers with aging might be influenced by the enrichment of longevity‐specific taxa or metabolites, since the growth of *Faecalibacterium* has been reported to be inhibited after incubation with isoalloLCA, generated by *Parabacteroides*, *Alistipes*, and other longevity‐related members (Sato et al., 2021). Correspondingly, *Parabacteroides* and *Alistipes* were also observed in higher abundance in our long‐living population than that in the general population, indicating a close relationship between the distribution of gut microbiota and metabolites.

Familial longevity represents a special group of long‐living individuals, and their specific fecal metabolomic and microbiota profiles confirmed this point of view. Here, we observed enrichment of *Parabacteroides* and PTA2 in a familial long‐living cohort. A possible mechanism by which *Parabacteroides* exerts its probiotic effects is the production of isoalloLCA and related bile acids, which play a role in protecting against potential pathogens (Sato et al., 2021). The source of PTA2 is unclear, but we suspected that it was influenced by longevity‐associated bacteria. Through correlation analysis, we found that PTA2 was significantly related to the abundance of *Alistipes*, *Ruminococcus_torques_group*, and *Erysipelotrichaceae_UCG‐003*, which have been reported to be highly enriched in long‐lived populations in previous studies (Biagi et al., 2016; Kong et al., 2016; Sato et al., 2021). In the aging profile, the abundance of PTA2 was positively correlated with age, and the linear regression plot showed a consistently higher abundance of PTA2 in individuals with familial longevity and their descendants than that in the general population in all age stages. Therefore, we speculated that PTA2 might play a key protective role during aging.

PTA2 is an analog of TXA2 and has been reported to play a role in inhibiting platelet aggregation (Byrne et al., 1997). In the central nervous system, the deposition of Aβ is closely related to senescence and can promote the degeneration of neurons, activation of microglia, and damage to the blood–brain barrier (Lau et al., 2020). Microglia, inherent phagocytes of the central nervous system, are responsible for the removal of Aβ. The clearance ability of microglia decreases with age and inflammation (Lana et al., 2021). Here, we pretreated microglial cells or organotypic slice cultures with PTA2 and then phagocytized with Aβ40. We observed that PTA2 played an anti‐inflammatory and protective role by enhancing the Aβ40‐specific phagocytic efficiency of microglia and reducing the production of TXA2 during phagocytosis by microglia. Specifically, STAT6 has been documented as a key molecule that mediates efferocytic activity and promotes inflammation resolution in microglia (Cai et al., 2019). Here, we observed activation of STAT6 signaling in microglia and inhibition of the aging‐related markers βgal and P16 by administration of PTA2. These observations indicate that PTA2 positively regulates phagocytosis and limits cellular inflammation in microglia after phagocytosis, resulting in delayed cellular senescence.

However, it is important to acknowledge that our study has several limitations. Firstly, the metabolite signatures were identified through untargeted metabolomic detection. While we have verified the functional role of these metabolites through in vitro experiments and we also evaluated the distribution of PTA2 in healthy people, further targeted metabolite analysis of PTA2 in both feces and blood is necessary. Secondly, the bacterial community at each age stage was characterized through 16S rRNA gene sequencing, which provides limited information for phylogenetic analysis. Nonetheless, functional genomics through metagenomic analysis could help elucidate the mechanisms underlying PTA2 generation. Thirdly, to bolster our research findings, an in vivo experiment to evaluate the protective role of PTA2 would be beneficial.

In conclusion, our study revealed fecal metabolomic and microbiota profiles in the longevity population, as well as their available trajectories with aging. We demonstrated that fecal metabolites, in addition to the previously reported microbiota, are promising biomarkers of aging. Additionally, we report that familial long‐living individuals harbor a distinct metabolomic and microbiota signature, especially enriched in PTA2, a beneficial compound with anti‐inflammatory and protective roles. Our study further reveals the importance of the gut microbiome in healthy aging and longevity.

## 4 EXPERIMENTAL PROCEDURES

### 4.1 Participants and study cohort

The study cohort was recruited from Jiaoling (the seventh longevity town of the world) in Meizhou City, situated in the northeast of Guangdong Province, China. In total, 155 participants were enrolled, consisting of 30 long‐living elderly (≥90 years, including 14 long‐living individuals associated with familial longevity) and 125 young‐age controls. Detailed information on the enrolled participants is presented in Table S1 and Figure S1. This study was approved by the Ethics Committee of the Third Affiliated Hospital of Sun Yat‐sen University (Ethics number: [2019]02‐010‐01), and informed consent was obtained from all study participants. The physical and cognitive health statuses of the enrolled participants were assessed using the Activities of Daily Living Scale and Mini‐Mental State Examination. The identification of familial longevity was based on the criteria from Marron et al. (2019): (1) long‐living individuals (proband) aged ≥90 years; (2) having at least one sibling who experienced longevity; (3) at least one enrolled offspring of the proband; and (4) the proband generation demonstrating clustering of exceptional survival (Family Longevity Selection Scores ≥7) (Sebastiani et al., 2009).

### 4.2 Fecal metabolomic analysis

#### 4.2.1 Sample preparation

Fecal samples were homogenized, and 100 mg of each fecal sample was dissolved in 800 μL methanol, vortexed, and centrifuged for 15 min at 10,000*g*. The supernatants were collected, vacuum dried, re‐dissolved in 200 μL of methanol/water (1:1), vortexed for 30 s, subjected to ultrasound for 10 min, centrifuged at 17,000*g* for 15 min, and filtered through a 0.22 μm filter. The resulting supernatant was stored at 4°C, and 10 μL of each supernatant was used for liquid chromatography and mass spectrometry (LC–MS) analysis.

### 4.3 Instrumentation and analytical conditions

Chromatographic analysis was performed on a ultra high performance liquid chromatography (UHPLC) system (1290, Agilent Technologies) equipped with an Acquity UPLC HSS T3 column (2.1 mm × 100 mm, 1.8 μm, Waters, USA) coupled to a quadrupole time of flight mass spectrometer (6545 Q‐TOF MS, Agilent Technologies, USA). Mobile phase A consisted of 0.1% formic acid in positive mode and 0.5 mmol/L ammonium fluoride in negative mode, and mobile phase B consisted of a 9:1 acetonitrile/water (v/v) solution with 0.1% formic acid. The flow rate was maintained at 0.3 mL/min with the following gradient: 0–4.0 min, 100% A, 4–6 min, 100% A, 6–25 min, 75% A, 25–29 min, 100% B, 29–31 min, 100% B, 31–33 min, 100% A. Parameters of mass spectrometry were set as follows: ion spray voltage, 4.0 kV (positive) or 3.5 kV (negative); curtain gas, 40 Pa; source temperature, 550°C; collision energy for collision‐induced dissociation, 30 eV. The MS1 scan range was 50–1000 *m/z*, and the MS2 scan range was 25–1000 *m/z*.

### 4.4 Metabolite identification and metabolomics data analysis

We used MS‐DIAL for peak search, peak alignment, and other data processing. The identification results were obtained based on database matching with the first‐ and second‐level maps. For mass spectrometry detection, data screening was performed under the following conditions: (1) using the QC sample to calculate the coefficient of variation (CV, RSD) to exclude peaks with a CV greater than 30% and (2) excluding peaks not detected in QC samples. MetaboAnalyst 5.0 (https://www.metaboanalyst.ca/MetaboAnalyst/home.xhtml) was used to analyze the datasets using pattern recognition methods.

### 4.5 Fecal microbiota analysis

Fecal DNA was extracted according to the operating instructions of the Trace Bacterial Flora DNA Extraction Kit I (LS‐R‐N‐007H‐50/100, Longsee). The V3‐V4 region of the 16S rRNA gene was PCR‐amplified by primers carrying Illumina overhang adapter sequences (forward: 338F: 5′‐ACTCCTACGGGAGGCAGCA‐3′; and reverse, 806R: 5′‐GGACTACHVGGGTWTCTAAT‐3′). Sequencing libraries were constructed, and paired‐end sequencing (2 × 250 bp) was performed using an Illumina MiSeq‐PE250 sequencer (San Diego, CA, USA). We used Custom Perl and Bash scripts to demultiplex the reads and assign barcoded reads to individual samples. Raw data were merged using FLASH. Trimmomatic was used for sequence qualification (Bolger et al., 2014), and the UCHIME algorithm was used to remove chimeric sequences (Edgar et al., 2011). The remaining sequences were binned into OTUs using USEARCH with a cutoff of 97% (Edgar, 2010). For single OTUs, the reads with the highest frequencies were chosen as representative sequences. The SILVA database was used for taxonomic assignments (Pruesse et al., 2007), and the Ribosomal Database Project, SeqMatch tool (http://rdp.cme.msu.edu), and Greengenes databases (http://greengenes.lbl.gov) were used for verification.

### 4.6 Functional analysis of PTA2 treatment on microglial cells

Mouse microglia were cultured in Dulbecco's modified Eagle's medium containing 10% fetal bovine serum (Pan Biotech) and 1% penicillin/streptomycin in a 10‐cm dish or 48‐wells plates at 37°C and 5% CO_2_. Cells were pretreated with 10 μM PTA2 for 2 h and then phagocytized with Aβ40 (20 μg/mL). Following stimulation, the supernatant and pellets were collected and immediately frozen at −20°C for subsequent analyses, including enzyme‐linked immunosorbent assay (ELISA), real‐time (RT)‐PCR, flow cytometry, and western blotting. Cells were fixed on coverslips and used for immunofluorescence staining.

### 4.7 Immunofluorescence staining

Mouse microglia were seeded on coverslips coated with poly‐l‐lysine (Sigma‐Aldrich). After treatment, the cells were fixed with 4% paraformaldehyde. Fixed cells were washed and permeabilized with 0.25% Triton X‐100 in phosphate‐buffered saline (PBS) for 20 min before being blocked in PBS containing 0.03% Triton‐X100 and 3% bovine serum albumin for 1 h, followed by overnight incubation with primary antibodies at 4°C. After three washes, cells were incubated with secondary antibodies for 1 h at room temperature. The following primary antibodies were used: rabbit anti‐A β40 (Invitrogen 44‐348A, 1:500) and sheep anti‐TREM2 (R&D Systems AF1729, 1:500). The following secondary antibodies were used: anti‐goat antibody conjugated with Cy3 (Jackson ImmunoResearch Laboratories 305‐165‐003, 1:1000) and anti‐rabbit antibody conjugated with Alexa Fluor 488 (Jackson ImmunoResearch Laboratories 112‐545‐003, 1:1000). The cells were washed and mounted with DAPI Fluoromount‐G (Southern Biotech).

### 4.8 Quantification of PTA2 in serum sample

#### 4.8.1 Sample preparation

Every collected 200 μL serum was purified by mixing with ice‐cold 800 μL Acetonitrile (ACN, HPLC grade), followed with centrifugation under 15,000 *g* for 10 min at 4°C. The supernatants were further freeze‐dried with the Alpha 3–4 LSCbasic (Christ, Germany) under default vacuum freeze‐drying program for organic solvent. Subsequently each dried sample was re‐suspended in 200 μL 20% ACN solvent (1:4 of acetonitrile: water). The above 200 μL sample was further purified with the IC C18 cartridge (Copure, China) based on manufactures' instruction and eluted to 100 μL solvent for further injection to mass spectrometry. Different concentrations (5 ng/μL to 1000 ng/μL) of PTA2 (Pinane thromboxane A2, GLPBIO) standards were also diluted with the same 20% ACN solvent.

### 4.9 Quantification of PTA2 by liquid chromatography–tandem mass spectrometry (LC–MS/MS)

The Nexera UHPLC system (Shimadzu, Japan) and Sciex 6500 Q TRAP® quadrupole ion trap mass spectrometer with an electrospray ionization (ESI) source (Applied Biosystems) were used for LC–MS/MS measurement. Samples were separated on an Acquity CSH C18 HPLC column (1.7 μm, 2.1 × 150 mm, Waters) at a flow rate of 0.2 mL/min. The mobile phase consisted of water (mobile phase A) and acetonitrile‐methanol (90:10; mobile phase B) using a gradient retention time of 0 min, 10% B; 6 min, 20% B; 12 min, 65% B; 16 min, 95% B; 20 min, 95% B; 23 min, 10% B. The column oven and auto‐sampler were maintained at 40°C and 4°C, respectively. The source temperature was set at 350°C, and the interface voltages were set at −4.5 kV for negative ion mode (MRM transition from 375.3 m/z to 275.1 m/z under the collision energy of −45 V). The injection volume for all samples was 10 μL. The area and concentration linear curve were analyzed through the Sciex OS software (Applied Biosystems) with 1/× weighting.

### 4.10 Organotypic slice cultures preparation, treatment, and immunostaining

The cerebellum was isolated from P7 Sprague Dawley rat pups and cut into 300 μm sections sagittally using a McIlwain tissue chopper. The sections were then plated onto Tissue Culture Plate Inserts (Corning 3460, Kennebunk, ME, USA) in 12‐well culture plates containing media. The media consisted of 50% Minimum Essential Medium (GIBCO, 11095080), 25% heat‐inactivated horse serum (GIBCO, 16050122), 25% Hanks' balanced salt solution (GIBCO, 14175‐095), 6.5 mg/mL d‐glucose (Sigma, G8769), 1% penicillin–streptomycin, and 2 mM/L‐glutamine. The slices were grown for 7 days in vitro in an incubator at 37°C, and the culture medium was changed every 2–3 days. PTA2 at 1 nM or 2 nM were added in the medium for 24 hours before TAMRA‐Amyloid‐β (1–40) peptides (25 mg/mL) treatment for phagocytosis. After 24 h, the culture medium was collected and the TAMRA fluorescence intensity was measured using a multimode microplate reader (Agilent BioTek Synergy H1MF). For immunostaining, slices were incubated with rabbit anti‐IBa1 antibody (Wako, 019‐197411:500) for 48 h at 4°C. Images were captured using confocal microscopy (Leica TSC SP8).

### 4.11 Flow cytometry

Mouse microglia were collected after treatment and washed with PBS. Next, cells were fixed, permeabilized (Invitrogen, Intracellular Fixation & Permeabilization Buffer Set), and stained with intracellular antibodies, followed by overnight incubation at 4°C in the dark. Fluorochrome compensation was performed using single‐stained OneComp eBeads (Thermo Fisher Scientific). Data analysis was performed using the FlowJo software (version 10.0).

### 4.12 ELISA

The supernatants of cultured microglia after treatment were collected by centrifugation (3000 rpm, 10 min) and stored at −80°C before examination of TXA2 concentration. A Mouse TXA2 ELISA Kit (HAKATA HZ‐030662) was used, and ELISA was performed according to the manufacturer's instructions.

### 4.13 Statistical analysis

Statistical tests were performed in R 3.3.2 using R Studio 1.0.136 and GraphPad Prism 8.0. The Wilcoxon rank‐sum test was used for comparisons between two groups, while one‐way ANOVA (using nonparametric tests) was used for comparisons among three or more groups. Spearman's correlations between discriminant fecal metabolites and bacteria were calculated using GraphPad Prism 8.0, and the clustering correlation heatmap with signs was constructed using the OmicStudio tools at https://www.omicstudio.cn. ROC analysis was conducted to evaluate the differentiated performance of fecal metabolites and bacteria, using OmicStudio tools (https://www.omicstudio.cn). Venn diagrams, linear regression plots, and distribution trends of the metabolites and bacteria on the age trajectory were plotted using Hiplot (https://hiplot.com.cn/basic/gene‐trend). For all analyses, a *p* value <0.05 was considered statistically significant.

## AUTHOR CONTRIBUTIONS

Z‐Q.L and Y‐J.L conceived and designed the experiments. J‐L.G, S‐X.L, and S‐S.W collected the questionnaires, worked on experiments, and analyzed the data. J‐L.G, S‐X.L, S‐S.W, Z‐Q.L, and Y‐J.L drafted the manuscript. Q‐Q. M helped with the targeted detection of PTA2 in serum samples. The rest of the authors helped to collect samples. All authors read and approved the final manuscript.

## CONFLICT OF INTEREST STATEMENT

The authors report no conflict of interest.

## Supporting information


Data S1

## Data Availability

The data supporting the findings of this study are available from the corresponding authors upon request. 16S rRNA sequencing data have been deposited in the National Center for Biotechnology Information (NCBI) database with the accession number PRJNA904535. Other data such as statistical analyses are provided in the supplementary materials with this paper.
