# Supplementary material for: Identification of fecal microbiome signatures associated with familial longevity and candidate metabolites for healthy aging
Source: Aging Cell. 2023 May 2;22(6):e13848. doi: 10.1111/acel.13848 (PMC10265179; doi:10.1111/acel.13848)
Supplement: Supplementary file 1 — Data S1 [file ACEL-22-e13848-s001.docx]

**Supporting information**

**Figure S1. Schematic representation of study design.**

**Figure S2. Principal coordinates analysis (A) and redundancy analysis (RDA) (B) of gut microbiota in group G, O, Y, and M.** G, grand elderly, n = 30; O, old elderly, n = 25; Y, young elderly, n = 75; M, middle and young age, n = 15. Each dot represents an individual.

**Figure S3. A multi-omics meta-analysis of metabolites and microbes together at the individual levels in** **group G, O, Y, and M.** Spearman’s correlation; r=0.4; p<0.05.

**Figure S4. Substructure in the distribution of microbiota and metabolites of individuals from G-FL** (A) Clustering heatmap between samples from G-FL; (B) Principal co-ordinates analysis (PCoA) showing the distribution of two subgroups in G-FL; (C) Variable influence on projection plot showing the top 20 most important metabolites in driving the separation of the two subgroups. (D) Partial least squares-discriminant analysis (PLS-DA) plot illustrating the distribution of fecal metabolites in the two subgroups. (E) Distance-based redundancy analysis (db-RDA) showing the relation between gut microbiota and eating habits as well as life styles.

**Table S1. Clinical characteristics of participants in this study**

**Table S2. Classification of the 20 most important metabolites into 6 clusters**

**Table S3. Classification of the 19 differentially distributed genera into 6 clusters**

**Table S4. Overlapped metabolites from Venn diagram**

**Table S5. Correlation analysis between 7 overlapped metabolites and the top 40 abundant genera in G-FL**

**Table S6. Investigations on the eating habits and life style of individuals of group G-FL**

**Table S7. Primers used in the study.**

**Experimental Procedures**

**Figure S1**

**
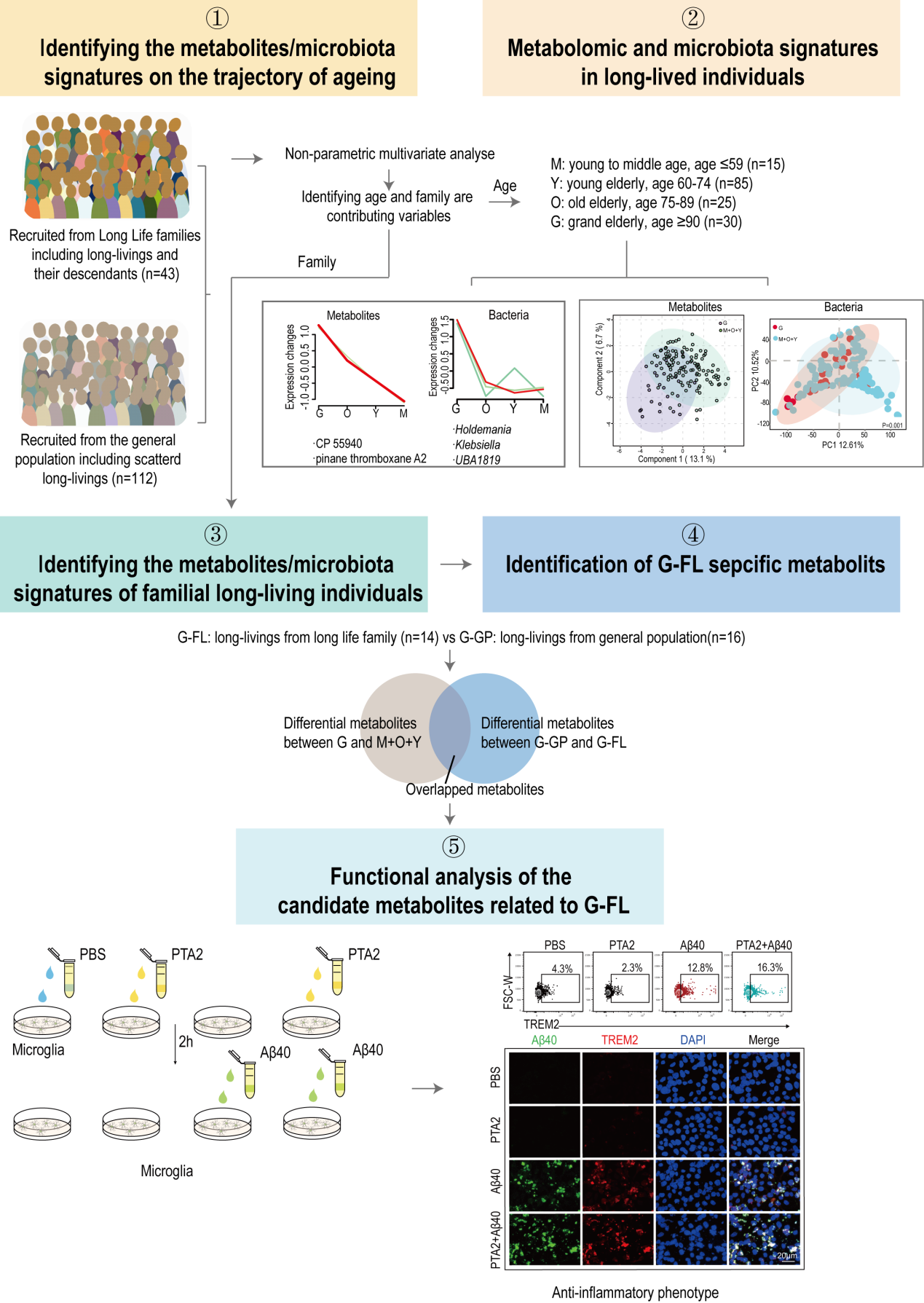
**

Figure S2

**
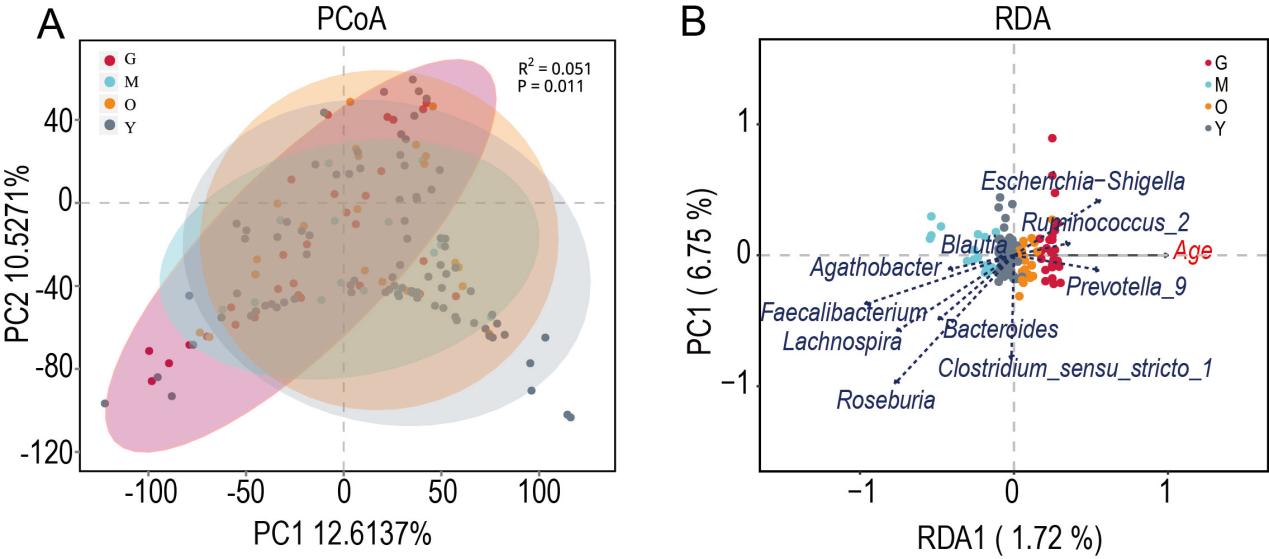
**

Figure S3


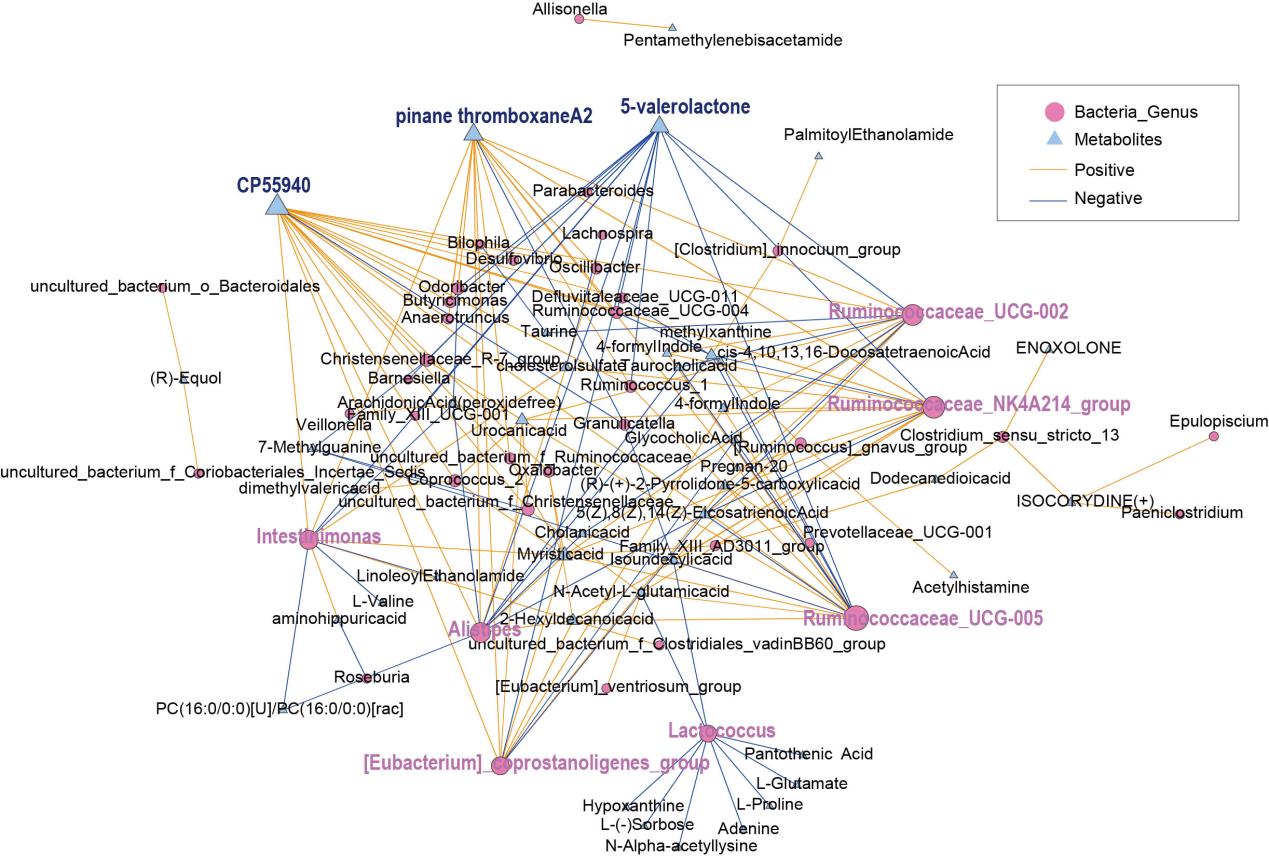


Figure S4

**
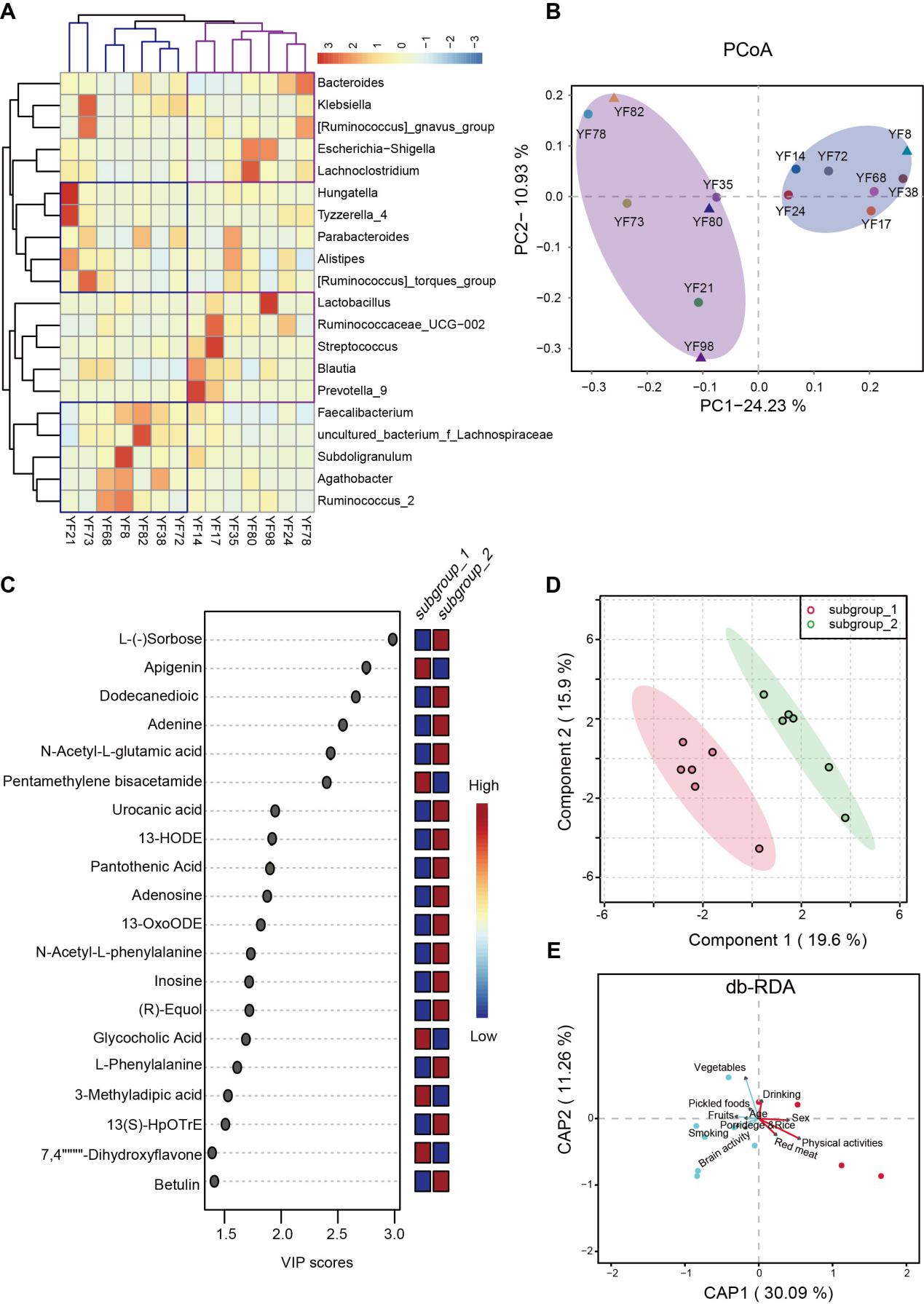
**

| **Supplementary Table S1. The basic characteristics of all participants in this study** | | | | | | | | | | |
| --- | --- | --- | --- | --- | --- | --- | --- | --- | --- | --- |
| **Subject** | **Familial longevity (FL) or Genenal population (GP)** | **Sex** | **Age** | **Group M (M):young to middle age (age≤59, n=15); Group Y (Y): young elderly (age 60-74, n=85); Group O (O), old elderly (age75-89, n=25); Group G (G), grand elderly (age≥90, n=30);** | **G-GP: (n=16) G-FL: (n=14)** | **Familial longlivings and their descendants: FL1 (longlivings, n=14); FL2 (the second generation, n=18); FL3 (the third generation, n=7); FL4 (the fourth generation, n=4);** | **MMSE** | **ADL** | **Education level** | **Disease (none:0, or with specific disease** |
| YF100 | FL | F | 10 | M |  | FL-4 | / | / | / | / |
| YF102 | GP | F | 101 | G | G-GP |  | / | 25 | illiteracy |  |
| YF103 | GP | F | 75 | O |  |  | 28 | 100 | illiteracy | 0 |
| YF104 | GP | F | 70 | Y |  |  | 30 | 100 | primary school | 0 |
| YF105 | GP | M | 79 | O |  |  | 28 | 100 | primary school | 0 |
| YF11 | FL | M | 53 | M |  | FL-2 | / | / | / | 0 |
| YF111 | GP | M | 61 | Y |  |  | 30 | 100 | junior school | 0 |
| YF112 | GP | M | 62 | Y |  |  | 23 | 100 | senior school | 0 |
| YF114 | GP | F | 78 | O |  |  | 18 | 100 | illiteracy | 0 |
| YF117 | GP | M | 84 | O |  |  | 18 | 100 | illiteracy | 0 |
| YF118 | GP | F | 95 | G | G-GP |  | 19 | 100 | illiteracy | cervical spondylosis |
| YF119 | GP | M | 63 | Y |  |  | 28 | 100 | junior school | gastritis |
| YF124 | GP | M | 68 | Y |  |  | 25 | 100 | primary school | hypertension |
| YF125 | GP | F | 68 | Y |  |  | 21 | 100 | illiteracy | 0 |
| YF126 | GP | F | 80 | O |  |  | 12 | 100 | illiteracy | 0 |
| YF127 | GP | M | 68 | Y |  |  | 25 | 100 | junior school | 0 |
| YF129 | GP | M | 67 | Y |  |  | 28 | 100 | primary school | hypertension |
| YF131 | GP | M | 61 | Y |  |  | 20 | 100 | senior school | 0 |
| YF135 | GP | M | 62 | Y |  |  | 30 | 100 | senior school | 0 |
| YF136 | GP | F | 69 | Y |  |  | 19 | 100 | illiteracy | 0 |
| YF137 | GP | M | 75 | O |  |  | 23 | 100 | primary school | 0 |
| YF14 | FL | F | 100 | G | G-FL | FL-1 | 10 | 85 | primary school | 0 |
| YF140 | GP | F | 60 | Y |  |  | 25 | 100 | primary school | 0 |
| YF141 | GP | M | 60 | Y |  |  | 29 | 100 | senior school | anemia, deafness |
| YF142 | GP | F | 66 | Y |  |  | 20 | 100 | illiteracy | 0 |
| YF143 | GP | F | 91 | G | G-GP |  | 18 | 100 | illiteracy | hypertension |
| YF144 | GP | M | 85 | O |  |  | 30 | 100 | illiteracy | hypertension |
| YF146 | GP | F | 61 | Y |  |  | 21 | 100 | primary school | 0 |
| YF147 | GP | F | 67 | Y |  |  | 30 | 100 | primary school | hyperlipidemia |
| YF149 | GP | F | 69 | Y |  |  | 13 | 95 | primary school | 0 |
| YF15 | FL | M | 37 | M |  | FL-3 | / | / | / |  |
| YF150 | GP | M | 77 | O |  |  | 23 | 100 | junior school | 0 |
| YF152 | GP | M | 64 | Y |  |  | 30 | 100 | junior school | 0 |
| YF154 | GP | F | 88 | O |  |  | 18 | 100 | illiteracy | 0 |
| YF155 | GP | M | 63 | Y |  |  | 28 | 100 | primary school | 0 |
| YF157 | GP | M | 62 | Y |  |  | 30 | 100 | primary school | 0 |
| YF158 | GP | M | 60 | Y |  |  | 30 | 100 | junior school | 0 |
| YF16 | GP | F | 97 | G | G-GP |  | / | / | / | 0 |
| YF163 | GP | M | 65 | Y |  |  | 27 | 100 | primary school | 0 |
| YF164 | GP | M | 63 | Y |  |  | 30 | 100 | senior school | 0 |
| YF165 | GP | F | 64 | Y |  |  | 25 | 100 | illiteracy | 0 |
| YF167 | GP | F | 78 | O |  |  | 21 | 100 | illiteracy | 0 |
| YF168 | GP | F | 81 | O |  |  | 20 | 100 | illiteracy | 0 |
| YF169 | GP | M | 60 | Y |  |  | 30 | 100 | primary school | 0 |
| YF17 | FL | M | 99 | G | G-FL | FL-1 | 11 | 100 | primary school | 0 |
| YF171 | GP | M | 70 | Y |  |  | 7 | 100 | primary school | 0 |
| YF172 | GP | M | 61 | Y |  |  | 28 | 100 | primary school | 0 |
| YF173 | GP | F | 72 | Y |  |  | 18 | 100 | illiteracy | hypertension; diabetes |
| YF174 | GP | M | 64 | Y |  |  | 30 | 100 | primary school | 0 |
| YF178 | GP | M | 73 | Y |  |  | 25 | 95 | junior school | diabetes |
| YF179 | GP | M | 68 | Y |  |  | 22 | 100 | illiteracy | colitis |
| YF182 | GP | F | 65 | Y |  |  | 15 | 100 | primary school | 0 |
| YF183 | GP | F | 91 | G | G-GP |  | 26 | 100 | illiteracy | 0 |
| YF187 | GP | M | 67 | Y |  |  | 29 | 100 | senior school | hypertension |
| YF188 | GP | F | 65 | Y |  |  | 16 | 100 | illiteracy | 0 |
| YF189 | GP | F | 60 | Y |  |  | 21 | 95 | primary school | 0 |
| YF19 | FL | M | 78 | O |  | FL-2 | / | / | / | / |
| YF191 | GP | M | 61 | Y |  |  | 30 | 100 | senior school | hypertension |
| YF192 | GP | F | 65 | Y |  |  | 29 | 100 | primary school | 0 |
| YF193 | GP | M | 68 | Y |  |  | 28 | 100 | senior school | hypertension |
| YF195 | GP | M | 63 | Y |  |  | 30 | 100 | primary school | 0 |
| YF196 | GP | F | 62 | Y |  |  | 27 | 100 | primary school | hypertension |
| YF198 | GP | F | 86 | O |  |  | 21 | 100 | illiteracy | hypertension |
| YF2 | FL | M | 84 | O |  | FL-2 | 6 | 100 | junior school | 0 |
| YF20 | GP | F | 72 | Y |  |  | / | / | / | / |
| YF200 | GP | F | 63 | Y |  |  | 24 | 100 | illiteracy | 0 |
| YF202 | GP | M | 61 | Y |  |  | 30 | 100 | junior school | 0 |
| YF203 | GP | M | 69 | Y |  |  | 25 | 100 | illiteracy | hypertension |
| YF204 | GP | M | 60 | Y |  |  | 29 | 100 | senior school | 0 |
| YF205 | GP | F | 79 | O |  |  | 18 | 100 | illiteracy | 0 |
| YF206 | GP | M | 66 | Y |  |  | 29 | 100 | senior school | cervical spondylosis |
| YF208 | GP | M | 84 | O |  |  | 19 | 100 | primary school | 0 |
| YF209 | GP | F | 64 | Y |  |  | 25 | 100 | primary school | 0 |
| YF21 | FL | F | 101 | G | G-FL | FL-1 | / | 90 | illiteracy | 0 |
| YF210 | GP | F | 71 | Y |  |  | 23 | 100 | primary school | 0 |
| YF213 | GP | M | 64 | Y |  |  | 30 | 100 | primary school | 0 |
| YF214 | GP | F | 62 | Y |  |  | 21 | 100 | illiteracy | 0 |
| YF216 | GP | F | 73 | Y |  |  | 14 | 100 | illiteracy | hypertension |
| YF217 | GP | M | 65 | Y |  |  | 19 | 100 | primary school | 0 |
| YF220 | GP | M | 68 | Y |  |  | / | / | / | / |
| YF221 | GP | M | 65 | Y |  |  | 23 | 100 | primary school | 0 |
| YF222 | GP | F | 75 | O |  |  | 17 | 100 | illiteracy | 0 |
| YF224 | GP | M | 60 | Y |  |  | 28 | 100 | junior school | 0 |
| YF225 | GP | F | 77 | O |  |  | 15 | 100 | illiteracy | 0 |
| YF228 | GP | F | 77 | O |  |  | 17 | 100 | illiteracy | 0 |
| YF23 | FL | F | 11 | M |  | FL-4 | / | / | / | 0 |
| YF230 | GP | F | 62 | Y |  |  | 30 | 100 | primary school | 0 |
| YF231 | GP | F | 62 | Y |  |  | 22 | 100 | junior school | hemorrhoid |
| YF232 | GP | M | 63 | Y |  |  | 27 | 100 | junior school | diabetes |
| YF234 | GP | M | 73 | Y |  |  | 26 | 100 | junior school | high uric acid |
| YF235 | GP | M | 65 | Y |  |  | 26 | 100 | primary school | 0 |
| YF237 | GP | F | 64 | Y |  |  | 23 | 100 | illiteracy | 0 |
| YF238 | GP | F | 68 | Y |  |  | 27 | 100 | illiteracy | 0 |
| YF239 | GP | M | 62 | Y |  |  | 24 | 100 | senior school | 0 |
| YF24 | FL | F | 101 | G | G-FL | FL-1 | / | 95 | illiteracy | 0 |
| YF240 | GP | F | 62 | Y |  |  | 28 | 100 | primary school | 0 |
| YF242 | GP | F | 83 | O |  |  | 21 | 100 | illiteracy | coronary heart disease |
| YF245 | GP | M | 100 | G | G-GP |  | 15 | 100 | illiteracy | 0 |
| YF246 | GP | F | 77 | O |  |  | 9 | 100 | junior school | 0 |
| YF247 | GP | F | 71 | Y |  |  | 26 | 100 | illiteracy | 0 |
| YF248 | GP | F | 78 | O |  |  | 23 | 100 | primary school | 0 |
| YF249 | GP | M | 69 | Y |  |  | / | / | / | 0 |
| YF25 | FL | M | 73 | Y |  | FL-2 | 24 | 100 | primary school | 0 |
| YF250 | GP | M | 66 | Y |  |  | 28 | 100 | primary school | gastritis |
| YF26 | GP | M | 71 | Y |  |  | 24 | 100 | primary school | 0 |
| YF27 | GP | F | 69 | Y |  |  | 21 | 100 | primary school | 0 |
| YF28 | FL | M | 42 | M |  | FL-3 | / | / | / | 0 |
| YF29 | FL | M | 58 | M |  | FL-2 | / | / | / | 0 |
| YF3 | FL | M | 72 | Y |  | FL-2 | 30 | 100 | junior school | 0 |
| YF34 | GP | F | 94 | G | G-GP |  | 18 | 90 | illiteracy | 0 |
| YF35 | FL | F | 99 | G | G-FL | FL-1 | / | 75 | illiteracy | 0 |
| YF36 | GP | F | 101 | G | G-GP |  | 0 | 65 | illiteracy | 0 |
| YF37 | GP | F | 64 | Y |  |  | 24 | 100 | primary school | 0 |
| YF38 | FL | F | 99 | G | G-FL | FL-1 | 26 | 100 | junior school | 0 |
| YF42 | GP | F | 105 | G | G-GP |  | 16 | 50 | illiteracy | 0 |
| YF43 | FL | M | 45 | M |  | FL-3 | / | / | / | 0 |
| YF45 | FL | F | 70 | Y |  | FL-2 | 28 | 100 | primary school | 0 |
| YF48 | GP | M | 63 | Y |  |  | 29 | 100 | senior school | 0 |
| YF49 | GP | F | 102 | G | G-GP |  | 19 | 90 | primary school | 0 |
| YF50 | FL | M | 50 | M |  | FL-2 | / | / | / | 0 |
| YF55 | FL | F | 61 | Y |  | FL-2 | 23 | 100 | junior school | 0 |
| YF58 | GP | F | 99 | G | G-GP |  | 21 | 55 | illiteracy | 0 |
| YF59 | GP | F | 72 | Y |  |  | 30 | 100 | primary school | 0 |
| YF6 | GP | F | 67 | Y |  |  | / | / | / | 0 |
| YF60 | FL | M | 70 | Y |  | FL-2 | / | / | / | / |
| YF61 | GP | M | 99 | G | G-GP |  | / | 35 | junior school | 0 |
| YF65 | GP | F | 85 | O |  |  | / | / | / | 0 |
| YF66 | FL | F | 50 | M |  | FL-3 | / | / | / | 0 |
| YF67 | FL | M | 50 | M |  | FL-3 | / | / | / | / |
| YF68 | FL | F | 99 | G | G-FL | FL-1 | 5 | 100 | illiteracy | 0 |
| YF69 | FL | M | 66 | Y |  | FL-2 | 29 | 75 | primary school | 0 |
| YF7 | GP | F | 99 | G | G-GP |  | 15 | 80 | illiteracy | 0 |
| YF70 | FL | M | 77 | O |  | FL-2 | 16 | 100 | illiteracy | 0 |
| YF72 | FL | F | 98 | G | G-FL | FL-1 | / | —— | primary school | 0 |
| YF73 | FL | M | 90 | G | G-FL | FL-1 | 30 | 100 | senior school | 0 |
| YF74 | FL | M | 11 | M |  | FL-4 | / | / | / | 0 |
| YF78 | FL | F | 103 | G | G-FL | FL-1 | 4 | 30 | primary school | 0 |
| YF79 | GP | M | 99 | G | G-GP |  | - | 15 | illiteracy | 0 |
| YF8 | FL | F | 101 | G | G-FL | FL-1 | 2 | 55 | illiteracy | 0 |
| YF80 | FL | F | 103 | G | G-FL | FL-1 | 17 | 60 | illiteracy | 0 |
| YF81 | FL | M | 65 | Y |  | FL-2 | 29 | 100 | primary school | 0 |
| YF82 | FL | M | 100 | G | G-FL | FL-1 | 22 | 75 | senior school | 0 |
| YF86 | FL | M | 61 | Y |  | FL-2 | 28 | 100 | junior school | 0 |
| YF87 | FL | F | 18 | M |  | FL-4 | / | / | / | 0 |
| YF88 | GP | F | 94 | G | G-GP |  | 27 | 100 | junior school | 0 |
| YF89 | GP | M | 100 | G | G-GP |  | / | 75 | senior school | 0 |
| YF9 | FL | M | 68 | Y |  | FL-2 | / | / | / | 0 |
| YF91 | FL | M | 55 | M |  | FL-2 | / | / | / | 0 |
| YF92 | FL | M | 69 | Y |  | FL-2 | 26 | 100 | primary school | 0 |
| YF93 | FL | M | 75 | O |  | FL-2 | 29 | 100 | junior school | 0 |
| YF94 | FL | M | 45 | M |  | FL-3 | / | / | / | 0 |
| YF95 | GP | F | 74 | Y |  |  | 15 | 100 | primary school | 0 |
| YF96 | FL | M | 42 | M |  | FL-3 | / | / | / | 0 |
| YF97 | GP | F | 64 | Y |  |  | 23 | 100 | senior school | 0 |
| YF98 | FL | F | 104 | G | G-FL | FL-1 | / | 45 | illiteracy | 0 |

| **Supplementary Table S2. Classification of the 20 most important metabolites into 6 clusters** | | | | | |
| --- | --- | --- | --- | --- | --- |
| **Metabolites** | **G** | **O** | **Y** | **M** | **Cluster** |
| cholanic acid | -1.12530257466549 | -0.46472424284604 | 0.438900467851144 | 1.15112634966038 | Cluster 1 |
| taurine | -1.2564348225137 | -0.322273196599462 | 0.600473069911473 | 0.978234949201687 | Cluster 1 |
| taurocholic acid | -1.20276723409907 | -0.332170643042606 | 0.403465847144238 | 1.13147202999744 | Cluster 1 |
| citrulline | -1.21292571324405 | 0.724591624247698 | 0.909202043946909 | -0.420867954950561 | Cluster 2 |
| 1-methylxanthine | -1.26907862554055 | 0.423050820846112 | 1.07592504561998 | -0.229897240925548 | Cluster 2 |
| inosine | -0.891003684197206 | 1.2055055701338 | 0.435797552938466 | -0.750299438875061 | Cluster 2 |
| L-tyrosine | -0.907380487258244 | 1.15212117432898 | 0.517679829908825 | -0.762420516979559 | Cluster 2 |
| PE(16:0/0:0) | -0.807592746309833 | 0.86407024938233 | 0.866071515111588 | -0.922549018184085 | Cluster 3 |
| 12,13-dihome | -0.802643531831464 | 0.701517721579772 | 1.01454501916353 | -0.913419208911833 | Cluster 3 |
| 3-methyladipic acid | -0.564914369463875 | -0.0785633342443373 | 1.43251960882212 | -0.789041905113903 | Cluster 3 |
| apigenin | -0.41818022788291 | 0.820640469352834 | 0.816288280594012 | -1.21874852206394 | Cluster 3 |
| PE(18:1(9Z)/0:0).1 | -0.625481907291884 | 0.976151105268211 | 0.717530721239215 | -1.06819991921554 | Cluster 3 |
| CP 55,940 | 1.28714596508392 | 0.199830917293639 | -0.429026404768542 | -1.05795047760902 | Cluster 4 |
| pinane thromboxane A2 | 1.21626604246643 | 0.329331300857404 | -0.442796978958841 | -1.10280036436499 | Cluster 4 |
| tyramine | 1.47482584823792 | -0.749507297951968 | -0.3561774547353 | -0.369141095550648 | Cluster 5 |
| aminohippuric acid | 1.14326212331701 | -1.13028098136346 | -0.462194622710742 | 0.449213480757192 | Cluster 5 |
| pentamethylene bisacetamide | 1.41827695682588 | -0.820210655405569 | -0.0374004845075128 | -0.560665816912797 | Cluster 5 |
| 3-amino-2-naphthoic acid | -1.40007482297271 | 0.787333866508222 | 0.647083064485409 | -0.0343421080209216 | Cluster 6 |
| 5-valerolactone | -1.46425185738404 | 0.538528797936745 | 0.725036700691432 | 0.200686358755859 | Cluster 6 |
| N-alpha-acetyllysine | -1.4052026945584 | 0.926425275675339 | 0.401449943824597 | 0.077327475058462 | Cluster 6 |

| **Supplementary Table S3. Classification of the 19 differentially distributed genera into 6 clusters** | | | | | |
| --- | --- | --- | --- | --- | --- |
| **Genus** | **G** | **O** | **Y** | **M** | **Cluster** |
| *Holdemania* | 1.48670231877124 | -0.317739078213942 | -0.637020009332955 | -0.531943231224347 | Cluster 1 |
| *Klebsiella* | 1.38361408746657 | -0.733699066331719 | 0.0849833203336144 | -0.734898341468462 | Cluster 1 |
| *UBA1819* | 1.49846591300983 | -0.457318857288613 | -0.562201490201805 | -0.478945565519415 | Cluster 1 |
| *Granulicatella* | -0.418399760502911 | -0.62894808836303 | -0.446093363191628 | 1.49344121205757 | Cluster 2 |
| *Lachnospira* | -0.65314486080143 | -0.536571890781977 | -0.293336260065847 | 1.48305301164925 | Cluster 2 |
| *Lactococcus* | -0.579978828888633 | -0.722230024599023 | -0.153300446612197 | 1.45550930009985 | Cluster 2 |
| *Butyricicoccus* | -1.23776614628161 | 0.353740575901896 | -0.247933577775106 | 1.13195914815482 | Cluster 3 |
| *Faecalibacterium* | -1.42732532578739 | 0.796868373857756 | 0.569202121244219 | 0.0612548306854142 | Cluster 3 |
| *Lachnospiraceae_*UCG-004 | -1.43655309802738 | 0.728881863991433 | 0.0757644596217645 | 0.631906774414182 | Cluster 3 |
| *Roseburia* | -1.35277459907793 | 1.03413542998022 | 0.317115871747755 | 0.00152329734995927 | Cluster 3 |
| *Ruminococcaceae_*UCG-013 | -1.31656682716159 | 0.454589551426188 | -0.155738606007045 | 1.01771588174244 | Cluster 3 |
| Family_XIII_AD3011_group | 1.37845560682432 | -0.0598069442905668 | -0.322526081695851 | -0.996122580837898 | Cluster 4 |
| *Mailhella* | 1.09687809011744 | 0.412658841512503 | -0.261199695548611 | -1.24833723608133 | Cluster 4 |
| *Negativibacillus* | -0.192089495755063 | 1.32541744460444 | -0.0355547091424813 | -1.0977732397069 | Cluster 4 |
| *uncultured_bacterium_f_Coriobacteriales_Incertae_Sedis* | -0.387748315146783 | 0.926086945684414 | 0.69184836733884 | -1.23018699787647 | Cluster 4 |
| *uncultured_bacterium_f_Ruminococcaceae* | 1.04580356988036 | 0.403535206985657 | -0.135957750665935 | -1.31338102620008 | Cluster 4 |
| *Anaerostipes* | 0.693312217601089 | -1.22588332090372 | -0.39504351258926 | 0.927614615891891 | Cluster 5 |
| *Fusicatenibacter* | -0.342935875518103 | -0.651369463018455 | -0.493753798267515 | 1.48805913680407 | Cluster 5 |
| *Intestinimonas* | 0.925352696748052 | 0.732435133378634 | -1.17031075497876 | -0.487477075147927 | Cluster 6 |

**Supplementary Table S4. Investigations on the eating habits and life style of individuals of group G-FL**

| **SampleId** | **Sex** | **Age** | **Porridge or rice** | **Red meat** | **Cereal**  **(250-400g)** | **Vegetables**  **(400-500g)** | **Fruits**  **(≥100g)** | **Pickled foods** | **High salt diet** | **Smoking** | **Drinking** | **Physical activities≥30min** | **Brain activity≥30min** | **Sleep**  **(6-8h)** |
| --- | --- | --- | --- | --- | --- | --- | --- | --- | --- | --- | --- | --- | --- | --- |
| **YF78** | 1 | 103 | 4 | 4 | 4 | 2 | 2 | 2 | 2 | 1 | 3 | 3 | 3 | 4 |
| **YF73** | 2 | 90 | 4 | 3 | 4 | 2 | 2 | 2 | 2 | 4 | 1 | 2 | 2 | 4 |
| **YF82** | 2 | 100 | 4 | 3 | 4 | 4 | 2 | 4 | 4 | 1 | 1 | 3 | 1 | 4 |
| **YF21** | 1 | 101 | 4 | 3 | 4 | 4 | 4 | 1 | 1 | 1 | 3 | 2 | 1 | 4 |
| **YF35** | 1 | 99 | 4 | 3 | 4 | 4 | 2 | 2 | 2 | 1 | 1 | 2 | 1 | 4 |
| **YF80** | 1 | 103 | 4 | 2 | 4 | 4 | 4 | 2 | 2 | 1 | 1 | 1 | 1 | 4 |
| **YF98** | 1 | 104 | 4 | 4 | 4 | 4 | 2 | 4 | 4 | 1 | 1 | 4 | 3 | 4 |
| **YF38** | 1 | 99 | 3 | 2 | 3 | 4 | 2 | 1 | 1 | 1 | 1 | 3 | 2 | 4 |
| **YF8** | 1 | 101 | 4 | 2 | 4 | 4 | 4 | 2 | 2 | 1 | 1 | 3 | 1 | 4 |
| **YF17** | 2 | 99 | 4 | 4 | 4 | 2 | 2 | 2 | 2 | 1 | 1 | 4 | 2 | 4 |
| **YF68** | 1 | 99 | 4 | 3 | 4 | 2 | 2 | 2 | 2 | 1 | 2 | 2 | 1 | 4 |
| **YF72** | 1 | 98 | 3 | 2 | 3 | 4 | 2 | 1 | 1 | 1 | 4 | 1 | 2 | 4 |
| **YF14** | 1 | 100 | 3 | 4 | 3 | 2 | 2 | 1 | 1 | 1 | 3 | 4 | 1 | 4 |
| **YF24** | 1 | 101 | 4 | 3 | 4 | 4 | 2 | 2 | 2 | 1 | 2 | 2 | 1 | 4 |

Note:

1: 0 days/week;

2: 1-3 days/week;

3: 4-6 days/week;

4: 7 days/week

| **Supplementary Table S5. Calculated overlapped metabolites from Venn diagram** | | |
| --- | --- | --- |
| **Data1** | **Data2** |  |
| **Differential metabolites between G and M+O+Y** | **Differential metabolites between G-GP and G-FL** | **Overlapped metabolites** |
| CP 55,940 | CP 55,940 | CP 55,940 |
| 3-amino-2-naphthoic acid | 3-amino-2-naphthoic acid | 3-amino-2-naphthoic acid |
| 5-valerolactone | 5-valerolactone | 5-valerolactone |
| Betulin | citrulline | isocorydine |
| isocorydine | isocorydine | PE(16:0/0:0) |
| PE(16:0/0:0) | L-tryptophan | pinane thromboxane a2 |
| pinane thromboxane a2 | PE(16:0/0:0) | inosine |
| dimethyl valeric acid | phenylacetaldehyde |  |
| 7-methylguanine | pinane thromboxane a2 |  |
| acacetin | yyramine |  |
| acetyl-DL-valine | 1-methylxanthine |  |
| adenosine | cholaninc acid |  |
| apigenin | aminohippuric acid |  |
| b-D-galactopyranose | glycocholic Acid |  |
| creatinine | inosine |  |
| eethyl-p-coumarat | L-tyrosine |  |
| inosine | pentamethylene bisacetamide |  |
| N-acetylhistamine | N-alpha-acetyllysine |  |
| PE(18:1(9Z)/0:0).1 | taurine |  |
| p-salicylic acid | taurocholic acid |  |

| **Supplementary Table S6. Correlation analysis between 7 overlapped metabolites and the top 40 abundant genera in G-FL** | | | | |
| --- | --- | --- | --- | --- |
| **Data1** | **Data2** | **rho** | **pvalue** | **relation** |
| Butyricicoccus | CP 55,940 | -0.8951049 | 5.94154E-06 | negtive |
| [Ruminococcus]_torques_group | Pinane Thromboxane A2 | 0.874125874 | 0.000308861 | positive |
| Butyricicoccus | Pinane Thromboxane A2 | -0.7972028 | 0.003161252 | negtive |
| uncultured_bacterium_f_Lachnospiraceae | PE(16:0/0:0) | 0.79020979 | 0.003616814 | positive |
| [Ruminococcus]_torques_group | CP 55,940 | 0.79020979 | 0.003616814 | positive |
| Alistipes | CP 55,940 | 0.783216783 | 0.00411545 | positive |
| Alistipes | Pinane Thromboxane A2 | 0.755244755 | 0.006596543 | positive |
| Blautia | isocorydine | -0.72027972 | 0.011020519 | negtive |
| Coprococcus_2 | isocorydine | -0.69015796 | 0.012986426 | negtive |
| Lachnospiraceae_UCG-004 | isocorydine | -0.68311553 | 0.014338998 | negtive |
| Sutterella | PE(16:0/0:0) | 0.679510674 | 0.015070229 | positive |
| [Ruminococcus]_torques_group | 3-amino-2-naphthoic acid | -0.66433566 | 0.022159207 | negtive |
| Megamonas | isocorydine | 0.660884295 | 0.019293714 | positive |
| Lachnospira | PE(16:0/0:0) | 0.657342657 | 0.023981083 | positive |
| Klebsiella | CP 55,940 | -0.65734266 | 0.023981083 | negtive |
| Parabacteroides | isocorydine | 0.65034965 | 0.025911517 | positive |
| Lactobacillus | 3-amino-2-naphthoic acid | 0.64778939 | 0.022740883 | positive |
| Blautia | Inosine | -0.64335664 | 0.027954553 | negtive |
| Tyzzerella_4 | PE(16:0/0:0) | -0.63636364 | 0.030114265 | negtive |
| Lactobacillus | Inosine | -0.6264337 | 0.029292738 | negtive |
| Butyricicoccus | PE(16:0/0:0) | 0.622377622 | 0.034800123 | positive |
| Phascolarctobacterium | PE(16:0/0:0) | -0.61538462 | 0.037334511 | negtive |
| Bacteroides | Inosine | 0.608391608 | 0.040002049 | positive |
| Erysipelotrichaceae_UCG-003 | Pinane Thromboxane A2 | 0.601844441 | 0.038406608 | positive |
| Ruminococcus_1 | isocorydine | -0.59440559 | 0.0457531 | negtive |
| Ruminococcaceae_UCG-002 | CP 55,940 | 0.584939601 | 0.045739074 | positive |
| [Eubacterium]_coprostanoligenes_group | isocorydine | -0.58041958 | 0.052086242 | negtive |
| Erysipelotrichaceae_UCG-003 | CP 55,940 | 0.572839889 | 0.051559177 | positive |
| Agathobacter | PE(16:0/0:0) | 0.566433566 | 0.059034148 | positive |
| Lachnoclostridium | isocorydine | 0.566433566 | 0.059034148 | positive |
| Faecalibacterium | PE(16:0/0:0) | 0.559440559 | 0.06274872 | positive |
| Sutterella | CP 55,940 | -0.55691854 | 0.059983608 | negtive |
| Alistipes | 3-amino-2-naphthoic acid | -0.55244755 | 0.066629001 | negtive |
| Megamonas | CP 55,940 | -0.55073691 | 0.063497906 | negtive |
| Sutterella | Pinane Thromboxane A2 | -0.54991328 | 0.06397667 | negtive |
| Bacteroides | isocorydine | 0.545454545 | 0.070678905 | positive |
| Klebsiella | PE(16:0/0:0) | 0.545454545 | 0.070678905 | positive |
| Phascolarctobacterium | Pinane Thromboxane A2 | 0.545454545 | 0.070678905 | positive |
| uncultured_bacterium_f_Lachnospiraceae | CP 55,940 | -0.53846154 | 0.074902287 | negtive |
| uncultured_bacterium_f_Lachnospiraceae | Pinane Thromboxane A2 | -0.53846154 | 0.074902287 | negtive |
| Intestinibacter | Pinane Thromboxane A2 | 0.531468531 | 0.079302939 | positive |
| Lactobacillus | isocorydine | -0.51253666 | 0.088416431 | negtive |
| Phascolarctobacterium | CP 55,940 | 0.503496503 | 0.09875157 | positive |
| Prevotella_2 | Pinane Thromboxane A2 | -0.50185881 | 0.096414404 | negtive |
| Erysipelotrichaceae_UCG-003 | 3-amino-2-naphthoic acid | -0.50032851 | 0.097599041 | negtive |
| Intestinibacter | CP 55,940 | 0.48951049 | 0.109632452 | positive |
| Megamonas | Inosine | 0.486484273 | 0.108761365 | positive |
| Escherichia-Shigella | Pinane Thromboxane A2 | 0.482517483 | 0.115373605 | positive |
| Klebsiella | Inosine | 0.482517483 | 0.115373605 | positive |
| Lachnospira | CP 55,940 | -0.48251748 | 0.115373605 | negtive |
| Lachnospira | Pinane Thromboxane A2 | -0.48251748 | 0.115373605 | negtive |
| [Eubacterium]_coprostanoligenes_group | Inosine | -0.47552448 | 0.121319356 | negtive |
| Faecalibacterium | 5-Valerolactone | 0.475524476 | 0.121319356 | positive |
| Faecalibacterium | Pinane Thromboxane A2 | -0.46853147 | 0.127472653 | negtive |
| Lachnospira | Inosine | 0.468531469 | 0.127472653 | positive |
| Romboutsia | CP 55,940 | 0.461538462 | 0.133836316 | positive |
| Ruminococcaceae_UCG-002 | isocorydine | -0.45884484 | 0.133506869 | negtive |
| Sutterella | Inosine | 0.44833694 | 0.143798117 | positive |
| Romboutsia | Pinane Thromboxane A2 | 0.447552448 | 0.147205378 | positive |
| Klebsiella | Pinane Thromboxane A2 | -0.44755245 | 0.147205378 | negtive |
| [Ruminococcus]_torques_group | PE(16:0/0:0) | -0.44755245 | 0.147205378 | negtive |
| Fusicatenibacter | isocorydine | -0.44483431 | 0.147338699 | negtive |
| Alistipes | PE(16:0/0:0) | -0.44055944 | 0.15421575 | negtive |
| Butyricicoccus | Inosine | 0.440559441 | 0.15421575 | positive |
| [Eubacterium]_eligens_group | Pinane Thromboxane A2 | 0.437829043 | 0.154586412 | positive |
| Prevotella_2 | CP 55,940 | -0.43067317 | 0.162220598 | negtive |
| Coprococcus_2 | Inosine | -0.42958812 | 0.163398621 | negtive |
| Ruminococcus_1 | PE(16:0/0:0) | 0.426573427 | 0.168899525 | positive |
| Intestinibacter | 3-amino-2-naphthoic acid | -0.42657343 | 0.168899525 | negtive |
| Tyzzerella_4 | 5-Valerolactone | -0.42657343 | 0.168899525 | negtive |
| Ruminococcaceae_UCG-002 | Pinane Thromboxane A2 | 0.423818513 | 0.169753407 | positive |
| Tyzzerella_4 | 3-amino-2-naphthoic acid | -0.41958042 | 0.176577011 | negtive |
| Megamonas | PE(16:0/0:0) | 0.413052684 | 0.182021679 | positive |
| Clostridium_sensu_stricto_1 | CP 55,940 | 0.412587413 | 0.184480685 | positive |
| Blautia | CP 55,940 | 0.405594406 | 0.192612184 | positive |
| [Eubacterium]_eligens_group | CP 55,940 | 0.402802719 | 0.194201669 | positive |
| Fusicatenibacter | CP 55,940 | 0.402802719 | 0.194201669 | positive |
| Phascolarctobacterium | 3-amino-2-naphthoic acid | -0.3986014 | 0.200972976 | negtive |
| Escherichia-Shigella | CP 55,940 | 0.398601399 | 0.200972976 | positive |
| Ruminococcus_2 | Pinane Thromboxane A2 | -0.39160839 | 0.209564352 | negtive |
| Lactobacillus | Pinane Thromboxane A2 | -0.3844025 | 0.21729649 | negtive |
| Escherichia-Shigella | PE(16:0/0:0) | -0.37762238 | 0.227443132 | negtive |
| Ruminococcus_1 | Pinane Thromboxane A2 | -0.37762238 | 0.227443132 | negtive |
| Dorea | 3-amino-2-naphthoic acid | -0.3677764 | 0.239527869 | negtive |
| Ruminococcus_2 | Inosine | -0.36363636 | 0.246255228 | negtive |
| Faecalibacterium | CP 55,940 | -0.35664336 | 0.256012537 | negtive |
| Clostridium_sensu_stricto_1 | Pinane Thromboxane A2 | 0.356643357 | 0.256012537 | positive |
| Streptococcus | 3-amino-2-naphthoic acid | -0.35664336 | 0.256012537 | negtive |
| uncultured_bacterium_f_Lachnospiraceae | 5-Valerolactone | 0.34965035 | 0.266004309 | positive |
| Lachnospira | isocorydine | 0.34965035 | 0.266004309 | positive |
| Anaerostipes | isocorydine | -0.34880967 | 0.266468445 | negtive |
| Megamonas | Pinane Thromboxane A2 | -0.34880004 | 0.266482545 | negtive |
| Dorea | Inosine | 0.343257969 | 0.27467141 | positive |
| Ruminococcus_2 | 3-amino-2-naphthoic acid | 0.342657343 | 0.276230513 | positive |
| Agathobacter | 3-amino-2-naphthoic acid | 0.335664336 | 0.286690914 | positive |
| Agathobacter | 5-Valerolactone | 0.335664336 | 0.286690914 | positive |
| Streptococcus | PE(16:0/0:0) | 0.335664336 | 0.286690914 | positive |
| Anaerostipes | Inosine | -0.33457254 | 0.287791292 | negtive |
| Bifidobacterium | isocorydine | -0.33217744 | 0.291470554 | negtive |
| Prevotella_9 | Inosine | 0.330994123 | 0.293298063 | positive |
| Megamonas | 5-Valerolactone | 0.330442147 | 0.294152738 | positive |
| [Eubacterium]_eligens_group | Inosine | 0.32924744 | 0.296007412 | positive |
| Parabacteroides | Pinane Thromboxane A2 | 0.328671329 | 0.297385078 | positive |
| Erysipelotrichaceae_UCG-003 | PE(16:0/0:0) | -0.3263012 | 0.300609187 | negtive |
| [Eubacterium]_eligens_group | 3-amino-2-naphthoic acid | -0.32574481 | 0.301482697 | negtive |
| Fusicatenibacter | Pinane Thromboxane A2 | 0.325744808 | 0.301482697 | positive |
| Ruminococcus_2 | PE(16:0/0:0) | 0.321678322 | 0.308312361 | positive |
| Roseburia | PE(16:0/0:0) | 0.315236911 | 0.318244998 | positive |
| Subdoligranulum | isocorydine | -0.31468531 | 0.319471913 | negtive |
| Lachnoclostridium | PE(16:0/0:0) | -0.31468531 | 0.319471913 | negtive |
| Faecalibacterium | Inosine | 0.314685315 | 0.319471913 | positive |
| Tyzzerella_4 | Pinane Thromboxane A2 | 0.314685315 | 0.319471913 | positive |
| [Ruminococcus]_torques_group | isocorydine | 0.307692308 | 0.330862674 | positive |
| Butyricicoccus | 3-amino-2-naphthoic acid | 0.307692308 | 0.330862674 | positive |
| Parabacteroides | Inosine | 0.307692308 | 0.330862674 | positive |
| Roseburia | 5-Valerolactone | -0.30472901 | 0.335507822 | negtive |
| Lachnospiraceae_UCG-004 | PE(16:0/0:0) | 0.302824411 | 0.338689908 | positive |
| Bacteroides | 3-amino-2-naphthoic acid | -0.3006993 | 0.342483375 | negtive |
| Blautia | 5-Valerolactone | -0.3006993 | 0.342483375 | negtive |
| Lachnospiraceae_UCG-004 | Inosine | -0.29578198 | 0.350596359 | negtive |
| Blautia | Pinane Thromboxane A2 | 0.293706294 | 0.354332534 | positive |
| Subdoligranulum | PE(16:0/0:0) | 0.293706294 | 0.354332534 | positive |
| Klebsiella | isocorydine | 0.293706294 | 0.354332534 | positive |
| Ruminococcus_1 | 3-amino-2-naphthoic acid | 0.293706294 | 0.354332534 | positive |
| uncultured_bacterium_f_Lachnospiraceae | Inosine | 0.286713287 | 0.366408456 | positive |
| Lachnoclostridium | Pinane Thromboxane A2 | 0.286713287 | 0.366408456 | positive |
| Bacteroides | CP 55,940 | -0.28671329 | 0.366408456 | negtive |
| Blautia | PE(16:0/0:0) | -0.27972028 | 0.378709234 | negtive |
| Lachnospiraceae_UCG-004 | Pinane Thromboxane A2 | -0.26761227 | 0.4003924 | negtive |
| Agathobacter | Pinane Thromboxane A2 | -0.26573427 | 0.403976659 | negtive |
| Escherichia-Shigella | 5-Valerolactone | -0.26573427 | 0.403976659 | negtive |
| Escherichia-Shigella | Inosine | -0.26573427 | 0.403976659 | negtive |
| Lachnoclostridium | Inosine | 0.265734266 | 0.403976659 | positive |
| Ruminococcus_1 | Inosine | -0.25874126 | 0.416938427 | negtive |
| Butyricicoccus | 5-Valerolactone | 0.258741259 | 0.416938427 | positive |
| Bifidobacterium | 3-amino-2-naphthoic acid | 0.253798491 | 0.426044388 | positive |
| Erysipelotrichaceae_UCG-003 | Inosine | 0.253789824 | 0.426060729 | positive |
| Subdoligranulum | 5-Valerolactone | 0.251748252 | 0.430115289 | positive |
| [Ruminococcus]_torques_group | Inosine | 0.251748252 | 0.430115289 | positive |
| Butyricicoccus | isocorydine | 0.251748252 | 0.430115289 | positive |
| Romboutsia | 3-amino-2-naphthoic acid | -0.24475524 | 0.443504276 | negtive |
| Tyzzerella_4 | CP 55,940 | 0.244755245 | 0.443504276 | positive |
| Ruminococcaceae_UCG-014 | Inosine | -0.2426015 | 0.447411028 | negtive |
| Ruminococcaceae_UCG-002 | Inosine | -0.238179 | 0.455988553 | negtive |
| Lachnospira | 5-Valerolactone | 0.237762238 | 0.457102207 | positive |
| Dorea | CP 55,940 | 0.234676367 | 0.462836644 | positive |
| Erysipelotrichaceae_UCG-003 | 5-Valerolactone | -0.23203641 | 0.468029724 | negtive |
| Bacteroides | 5-Valerolactone | -0.23076923 | 0.470905694 | negtive |
| uncultured_bacterium_f_Lachnospiraceae | isocorydine | 0.230769231 | 0.470905694 | positive |
| Ruminococcus_2 | isocorydine | -0.23076923 | 0.470905694 | negtive |
| [Ruminococcus]_torques_group | 5-Valerolactone | -0.23076923 | 0.470905694 | negtive |
| Anaerostipes | CP 55,940 | 0.227794071 | 0.476431266 | positive |
| Dorea | Pinane Thromboxane A2 | 0.227671102 | 0.476675824 | positive |
| Prevotella_9 | 5-Valerolactone | 0.225357701 | 0.481287413 | positive |
| Prevotella_2 | isocorydine | -0.22067551 | 0.490683004 | negtive |
| Lactobacillus | CP 55,940 | -0.22067551 | 0.490683004 | negtive |
| Ruminococcaceae_UCG-014 | 5-Valerolactone | 0.220207514 | 0.491626634 | positive |
| [Eubacterium]_eligens_group | PE(16:0/0:0) | 0.210157941 | 0.512085329 | positive |
| Ruminococcus_2 | CP 55,940 | -0.20979021 | 0.513512513 | negtive |
| Ruminococcus_1 | CP 55,940 | -0.20979021 | 0.513512513 | negtive |
| Phascolarctobacterium | isocorydine | 0.20979021 | 0.513512513 | positive |
| Ruminococcaceae_UCG-014 | CP 55,940 | 0.209010522 | 0.514444662 | positive |
| Prevotella_9 | PE(16:0/0:0) | 0.204230416 | 0.524324327 | positive |
| Dorea | PE(16:0/0:0) | 0.203152676 | 0.526563053 | positive |
| Ruminococcaceae_UCG-002 | PE(16:0/0:0) | -0.20315268 | 0.526563053 | negtive |
| [Ruminococcus]_gnavus_group | PE(16:0/0:0) | -0.2027972 | 0.528100237 | negtive |
| Streptococcus | CP 55,940 | -0.2027972 | 0.528100237 | negtive |
| Streptococcus | 5-Valerolactone | -0.2027972 | 0.528100237 | negtive |
| Alistipes | 5-Valerolactone | -0.1958042 | 0.542873521 | negtive |
| Prevotella_2 | 5-Valerolactone | -0.19576053 | 0.542027792 | negtive |
| Lactobacillus | 5-Valerolactone | 0.192201248 | 0.549541122 | positive |
| Roseburia | Inosine | 0.189142146 | 0.55603285 | positive |
| Prevotella_9 | isocorydine | 0.183103132 | 0.568939592 | positive |
| Clostridium_sensu_stricto_1 | PE(16:0/0:0) | 0.181818182 | 0.572958221 | positive |
| [Ruminococcus]_gnavus_group | isocorydine | 0.181818182 | 0.572958221 | positive |
| Streptococcus | isocorydine | -0.18181818 | 0.572958221 | negtive |
| Lachnospiraceae_UCG-004 | 3-amino-2-naphthoic acid | 0.176060704 | 0.58414053 | positive |
| Alistipes | isocorydine | 0.174825175 | 0.588259895 | positive |
| Streptococcus | Inosine | 0.174825175 | 0.588259895 | positive |
| Clostridium_sensu_stricto_1 | 3-amino-2-naphthoic acid | -0.17482517 | 0.588259895 | negtive |
| Clostridium_sensu_stricto_1 | isocorydine | -0.17482517 | 0.588259895 | negtive |
| Streptococcus | Pinane Thromboxane A2 | -0.17482517 | 0.588259895 | negtive |
| Megamonas | 3-amino-2-naphthoic acid | 0.165221074 | 0.607841489 | positive |
| Fusicatenibacter | Inosine | -0.16112109 | 0.616898856 | negtive |
| Faecalibacterium | 3-amino-2-naphthoic acid | 0.160839161 | 0.619356169 | positive |
| uncultured_bacterium_f_Lachnospiraceae | 3-amino-2-naphthoic acid | 0.160839161 | 0.619356169 | positive |
| Faecalibacterium | isocorydine | 0.153846154 | 0.635139955 | positive |
| [Eubacterium]_coprostanoligenes_group | CP 55,940 | 0.153846154 | 0.635139955 | positive |
| Klebsiella | 5-Valerolactone | -0.14685315 | 0.651073351 | negtive |
| [Eubacterium]_coprostanoligenes_group | 5-Valerolactone | -0.14685315 | 0.651073351 | negtive |
| Ruminococcaceae_UCG-014 | isocorydine | -0.1455609 | 0.651712524 | negtive |
| Agathobacter | CP 55,940 | -0.13986014 | 0.667150536 | negtive |
| Prevotella_2 | PE(16:0/0:0) | 0.138812012 | 0.667017986 | positive |
| Ruminococcaceae_UCG-014 | PE(16:0/0:0) | 0.138096238 | 0.66864823 | positive |
| Roseburia | Pinane Thromboxane A2 | 0.136602661 | 0.672054217 | positive |
| Subdoligranulum | CP 55,940 | 0.132867133 | 0.683365534 | positive |
| Alistipes | Inosine | 0.132867133 | 0.683365534 | positive |
| [Ruminococcus]_gnavus_group | CP 55,940 | -0.13286713 | 0.683365534 | negtive |
| Parabacteroides | CP 55,940 | 0.132867133 | 0.683365534 | positive |
| [Eubacterium]_eligens_group | 5-Valerolactone | -0.1295974 | 0.688103944 | negtive |
| Ruminococcaceae_UCG-002 | 3-amino-2-naphthoic acid | -0.1295974 | 0.688103944 | negtive |
| Prevotella_2 | 3-amino-2-naphthoic acid | 0.128134165 | 0.691471563 | positive |
| Coprococcus_2 | 5-Valerolactone | -0.12676371 | 0.694630326 | negtive |
| Parabacteroides | 3-amino-2-naphthoic acid | -0.12587413 | 0.699712221 | negtive |
| Bifidobacterium | Pinane Thromboxane A2 | -0.12316691 | 0.70294168 | negtive |
| Roseburia | isocorydine | -0.12259213 | 0.704272667 | negtive |
| Coprococcus_2 | Pinane Thromboxane A2 | -0.11972128 | 0.71093186 | negtive |
| Agathobacter | isocorydine | -0.11888112 | 0.716184327 | negtive |
| Agathobacter | Inosine | -0.11888112 | 0.716184327 | negtive |
| [Eubacterium]_coprostanoligenes_group | 3-amino-2-naphthoic acid | 0.118881119 | 0.716184327 | positive |
| Tyzzerella_4 | Inosine | -0.11888112 | 0.716184327 | negtive |
| Fusicatenibacter | 5-Valerolactone | -0.11558687 | 0.720554564 | negtive |
| Ruminococcus_2 | 5-Valerolactone | 0.111888112 | 0.732775446 | positive |
| [Ruminococcus]_gnavus_group | 3-amino-2-naphthoic acid | 0.111888112 | 0.732775446 | positive |
| [Ruminococcus]_gnavus_group | 5-Valerolactone | 0.111888112 | 0.732775446 | positive |
| Blautia | 3-amino-2-naphthoic acid | -0.11188811 | 0.732775446 | negtive |
| Coprococcus_2 | CP 55,940 | 0.105636422 | 0.743864672 | positive |
| Anaerostipes | 5-Valerolactone | 0.099659906 | 0.75796219 | positive |
| Intestinibacter | 5-Valerolactone | -0.0979021 | 0.766288456 | negtive |
| Prevotella_9 | CP 55,940 | 0.091551566 | 0.777196289 | positive |
| Bacteroides | PE(16:0/0:0) | -0.09090909 | 0.78319691 | negtive |
| Lachnoclostridium | CP 55,940 | 0.090909091 | 0.78319691 | positive |
| Roseburia | CP 55,940 | 0.087565809 | 0.786694014 | positive |
| Clostridium_sensu_stricto_1 | 5-Valerolactone | 0.083916084 | 0.800197518 | positive |
| Parabacteroides | PE(16:0/0:0) | -0.08391608 | 0.800197518 | negtive |
| Intestinibacter | Inosine | 0.083916084 | 0.800197518 | positive |
| Romboutsia | PE(16:0/0:0) | -0.08391608 | 0.800197518 | negtive |
| Lactobacillus | PE(16:0/0:0) | 0.081863494 | 0.80032839 | positive |
| Prevotella_9 | Pinane Thromboxane A2 | 0.07746671 | 0.810876457 | positive |
| Bifidobacterium | 5-Valerolactone | -0.07464661 | 0.817657322 | negtive |
| Sutterella | 5-Valerolactone | -0.07355528 | 0.820284526 | negtive |
| Coprococcus_2 | PE(16:0/0:0) | 0.070424282 | 0.827831199 | positive |
| Lachnospiraceae_UCG-004 | CP 55,940 | -0.07042428 | 0.827831199 | negtive |
| Dorea | isocorydine | 0.070052647 | 0.828727854 | positive |
| Klebsiella | 3-amino-2-naphthoic acid | -0.06993007 | 0.834447145 | negtive |
| [Eubacterium]_coprostanoligenes_group | PE(16:0/0:0) | 0.06993007 | 0.834447145 | positive |
| Romboutsia | 5-Valerolactone | -0.06993007 | 0.834447145 | negtive |
| Romboutsia | Inosine | 0.06993007 | 0.834447145 | positive |
| Bifidobacterium | CP 55,940 | -0.06344962 | 0.844689572 | negtive |
| Lachnospira | 3-amino-2-naphthoic acid | 0.062937063 | 0.851681911 | positive |
| [Eubacterium]_eligens_group | isocorydine | 0.05954475 | 0.854154625 | positive |
| Bifidobacterium | Inosine | -0.05598496 | 0.862798664 | negtive |
| Escherichia-Shigella | 3-amino-2-naphthoic acid | 0.055944056 | 0.868980339 | positive |
| Clostridium_sensu_stricto_1 | Inosine | 0.055944056 | 0.868980339 | positive |
| Phascolarctobacterium | 5-Valerolactone | 0.055944056 | 0.868980339 | positive |
| Roseburia | 3-amino-2-naphthoic acid | -0.05253949 | 0.871178309 | negtive |
| [Ruminococcus]_gnavus_group | Pinane Thromboxane A2 | -0.04895105 | 0.886335109 | negtive |
| Parabacteroides | 5-Valerolactone | -0.04895105 | 0.886335109 | negtive |
| Ruminococcaceae_UCG-014 | Pinane Thromboxane A2 | 0.044787969 | 0.890073873 | positive |
| Lachnoclostridium | 5-Valerolactone | -0.04195804 | 0.903738836 | negtive |
| [Ruminococcus]_gnavus_group | Inosine | 0.041958042 | 0.903738836 | positive |
| Romboutsia | isocorydine | 0.041958042 | 0.903738836 | positive |
| Anaerostipes | PE(16:0/0:0) | 0.035592824 | 0.912556071 | positive |
| Lachnospiraceae_UCG-004 | 5-Valerolactone | 0.035212141 | 0.91348822 | positive |
| Bacteroides | Pinane Thromboxane A2 | -0.03496503 | 0.921184083 | negtive |
| Subdoligranulum | Pinane Thromboxane A2 | -0.03496503 | 0.921184083 | negtive |
| Intestinibacter | isocorydine | -0.03496503 | 0.921184083 | negtive |
| Sutterella | 3-amino-2-naphthoic acid | 0.031523691 | 0.922524865 | positive |
| Anaerostipes | Pinane Thromboxane A2 | 0.028474259 | 0.930002326 | positive |
| Tyzzerella_4 | isocorydine | 0.027972028 | 0.938663363 | positive |
| Lachnoclostridium | 3-amino-2-naphthoic acid | -0.02797203 | 0.938663363 | negtive |
| Erysipelotrichaceae_UCG-003 | isocorydine | -0.02175341 | 0.946499972 | negtive |
| Coprococcus_2 | 3-amino-2-naphthoic acid | 0.021127284 | 0.948038005 | positive |
| Escherichia-Shigella | isocorydine | -0.02097902 | 0.956169155 | negtive |
| Subdoligranulum | 3-amino-2-naphthoic acid | 0.020979021 | 0.956169155 | positive |
| Sutterella | isocorydine | -0.01751316 | 0.956918824 | negtive |
| Ruminococcaceae_UCG-002 | 5-Valerolactone | -0.01751316 | 0.956918824 | negtive |
| Fusicatenibacter | PE(16:0/0:0) | -0.01401053 | 0.965529985 | negtive |
| Phascolarctobacterium | Inosine | 0.013986014 | 0.973693904 | positive |
| Intestinibacter | PE(16:0/0:0) | 0.013986014 | 0.973693904 | positive |
| Subdoligranulum | Inosine | -0.01398601 | 0.973693904 | negtive |
| Prevotella_2 | Inosine | -0.01067785 | 0.97372648 | negtive |
| Bifidobacterium | PE(16:0/0:0) | 0.007464661 | 0.981631299 | positive |
| Anaerostipes | 3-amino-2-naphthoic acid | 0.007118565 | 0.982482841 | positive |
| Ruminococcus_1 | 5-Valerolactone | -0.00699301 | 0.991230036 | negtive |
| Fusicatenibacter | 3-amino-2-naphthoic acid | -0.00350263 | 0.991380382 | negtive |

| **Supplementary Table S7. Primers used in the study.** | | |
| --- | --- | --- |
| **Gene** | **Forwards primer** | **Reverse primer** |
| *Tnfa* | CTGAACTTCGGGGTGATCGG | GGCTTGTCACTCGAATTTTGAGA |
| *Il10* | CTTACTGACTGGCATGAGGATCA | GCAGCTCTAGGAGCATGTGG |
| *Arg1* | CTCCAAGCCAAAGTCCTTAGAG | GGAGCTGTCATTAGGGACATCA |
| *Trem2* | ACAGCACCTCCAGGAATCAAG | AACTTGCTCAGGAGAACGCA |
| *Gapdh* | CCCTTAAGAGGGATGCTGCC | TACGGCCAAATCCGTTCACA |
| *Il1b* | GAAATGCCACCTTTTGACAGTG | TGGATGCTCTCATCAGGACAG |
| *Il6* | CTGCAAGAGACTTCCATCCAG | AGTGGTATAGACAGGTCTGTTGG |
| *Tgfb* | GAGCCAGAACGAGAAGTACCG | CCTCAAGACGAGCAATTTCATCA |
| *Il17a* | CCTGGACTCTCCACCGCAA | TTCCCTCCGCATTGACACAG C |
| *Il4* | GGTCTCAACCCCCAGCTAGT | GCCGATGATCTCTCTCAAGTGAT |

**Experimental Procedures**

**Participants and study cohort**

The study cohort was recruited from Jiaoling (the seventh longevity town of the world) in Meizhou City, situated in the northeast of Guangdong Province, China. In total, 155 participants were enrolled, consisting of 30 long-living elderly (≥90 years, including 14 long-living individuals associated with familial longevity) and 125 young-age controls. All the elderly or group G are from retirement homes. Detailed information on the enrolled participants is presented in Table S1 and Figure S1. This study was approved by the Ethics Committee of the Third Affiliated Hospital of Sun Yat-sen University (Ethics number: [2019]02-010-01), and informed consent was obtained from all study participants. The physical and cognitive health statuses of the enrolled participants were assessed using the Activities of Daily Living Scale and Mini-Mental State Examination. The identification of familial longevity was based on the criteria from Marron et al. (2019): (1) long-living individuals (proband) aged ≥90 years; (2) having at least one sibling who experienced longevity; (3) at least one enrolled offspring of the proband; and (4) the proband generation demonstrating clustering of exceptional survival (Family Longevity Selection Scores ≥7) (Sebastiani et al., 2009).

**Fecal metabolomic analysis**

**Sample preparation**

Fecal samples were homogenized, and 100 mg of each fecal sample was dissolved in 800 μL methanol, vortexed, and centrifuged for 15 min at 10,000 × *g.* The supernatants were collected, vacuum dried, re-dissolved in 200 µL of methanol/water (1:1), vortexed for 30 s, subjected to ultrasound for 10 min, centrifuged at 17,000 *g* for 15 min, and filtered through a 0.22 μm filter. The resulting supernatant was stored at 4 °C, and 10 μL of each supernatant was used for liquid chromatography and mass spectrometry (LC-MS) analysis.

**Instrumentation and analytical conditions**

Chromatographic analysis was performed on a UHPLC system (1290, Agilent Technologies) equipped with an Acquity UPLC HSS T3 column (2.1 mm × 100 mm, 1.8 μm, Waters, USA) coupled to a quadrupole time of flight mass spectrometer (6545 Q-TOF MS, Agilent Technologies, USA). Mobile phase A consisted of 0.1% formic acid in positive mode and 0.5 mmol/L ammonium fluoride in negative mode, and mobile phase B consisted of a 9:1 acetonitrile/water (v/v) solution with 0.1% formic acid. The flow rate was maintained at 0.3 mL/min with the following gradient: 0-4.0 min, 100% A, 4-6 min, 100% A, 6-25 min, 75% A, 25-29 min, 100% B, 29-31 min, 100% B, 31-33 min, 100% A. Parameters of mass spectrometry were set as follows: ion spray voltage, 4.0 kV (positive) or 3.5 kV (negative); curtain gas, 40 Pa; source temperature, 550 °C; collision energy for collision-induced dissociation, 30 eV. The MS1 scan range was 50-1,000 *m/z,* and the MS2 scan range was 25-1,000 *m/z.*

**Metabolite identification and metabolomics data analysis**

We used MS-DIAL for peak search, peak alignment, and other data processing. The identification results were obtained based on database matching with the first- and second-level maps. For mass spectrometry detection, data screening was performed under the following conditions: (1) using the QC sample to calculate the coefficient of variation (CV, RSD) to exclude peaks with a CV greater than 30% and (2) excluding peaks not detected in QC samples. MetaboAnalyst 5.0 (<https://www.metaboanalyst.ca/MetaboAnalyst/home.xhtml>) was used to analyze the datasets using pattern recognition methods. To reduce the concentration variance among samples, the data were normalized to the total spectral peak height and log-transformed. For multivariate statistical analysis, PLS-DA was employed to eliminate intersubject variability among participants. Metabolites were ranked based on VIP scores from the PLS-DA model. Metabolites with VIP scores >1.0 were regarded as significant contributors.

**Fecal microbiota analysis**

**DNA extraction and 16S rRNA gene sequencing**

Fecal DNA was extracted according to the operating instructions of the Trace Bacterial Flora DNA Extraction Kit I (LS-R-N-007H-50/100, Longsee). DNA concentrations were evaluated using a NanoDrop ND-1000 spectrophotometer (NanoDrop Technologies, Wilmington, DE, USA). The V3-V4 region of the 16S rRNA gene was PCR-amplified in 20 μL volumes containing 20 ng of DNA, 10 μL of 2× Phanta Max Master Mix (Vazyme Biotech Co., Ltd), and 1 μL of each primer (10 μmol/L) (forward, 338F: 5’-ACTCCTACGGGAGGCAGCA-3’; and reverse, 806R: 5’-GGACTACHVGGGTWTCTAAT-3’) carrying Illumina overhang adapter sequences. The PCR thermal cycling scheme was set as follows: initial denaturation at 95 ℃ for 3 min; 30 cycles of denaturation at 95 ℃ for 15 s, annealing at 55 ℃ for 15 s, and extension at 72 ℃ for 30 s; followed by a final extension period at 72 ℃ for 5 min. PCR products were purified using a magnetic bead-based clean-up system (Agencourt AMPure XP; Beckman Coulter, Brea, CA, USA). Sequencing libraries were constructed, and paired-end sequencing (2 × 250 bp) was performed using an Illumina MiSeq-PE250 sequencer (San Diego, CA, USA).

**Sequence assembly and data analysis**

We used Custom Perl and Bash scripts to demultiplex the reads and assign barcoded reads to individual samples. Reads were filtered out when the sequences did not match the barcode and the V3-V4 16S rRNA gene primers or the length did not match the expected length for the V3-V4 variable region. Raw data were merged using FLASH. Trimmomatic was used for sequence qualification (Bolger et al., 2014), and the UCHIME algorithm was used to remove chimeric sequences (Edgar et al., 2011). The remaining sequences were binned into OTUs using USEARCH with a cutoff of 97% (Edgar et al., 2010). For single OTUs, the reads with the highest frequencies were chosen as representative sequences. The SILVA database was used for taxonomic assignments (Pruesse et al., 2007), and the Ribosomal Database Project, SeqMatch tool (<http://rdp.cme.msu.edu>), and Greengenes databases (<http://greengenes.lbl.gov>) were used for verification. Taxonomy was assigned based on hits with the highest percentage of identity coverage. When multiple hits fulfilled this criterion, the classification was reassigned to a more common taxonomy. The QIIME pipeline was used for (i) taxonomic distribution, (ii) PCoA, and (iii) LDA effect size analyses. Correlation network analysis was performed using OmicStudio tools at <https://www.omicstudio.cn/tool>.

**Functional analysis of PTA2 treatment on microglial cells**

Mouse microglia were cultured in Dulbecco’s modified Eagle’s medium containing 10% fetal bovine serum (Pan Biotech) and 1% penicillin/streptomycin in a 10-cm dish or 48-wells plates at 37 °C and 5% CO_2_. Cells were pretreated with 10 μM PTA2 for 2 h and then phagocytized with Aβ40 (20 μg/mL). Following stimulation, the supernatant and pellets were collected and immediately frozen at -20 °C for subsequent analyses, including enzyme-linked immunosorbent assay (ELISA), real-time (RT)-PCR, flow cytometry, and western blotting. Cells were fixed on coverslips and used for immunofluorescence staining.

**Immunofluorescence staining**

Mouse microglia were seeded on coverslips coated with poly-l-lysine (Sigma-Aldrich). After treatment, the cells were fixed with 4% paraformaldehyde. Fixed cells were washed and permeabilized with 0.25% Triton X-100 in phosphate-buffered saline (PBS) for 20 min before being blocked in PBS containing 0.03% Triton-X100 and 3% bovine serum albumin for 1 h, followed by overnight incubation with primary antibodies at 4 °C. After three washes, cells were incubated with secondary antibodies for 1 h at room temperature. The following primary antibodies were used: rabbit anti-Aβ40 (Invitrogen 44-348A, 1:500) and sheep anti-TREM2 (R&D Systems AF1729, 1:500). The following secondary antibodies were used: anti-goat antibody conjugated with Cy3 (Jackson ImmunoResearch Laboratories 305-165-003, 1:1,000) and anti-rabbit antibody conjugated with Alexa Fluor 488 (Jackson ImmunoResearch Laboratories 112-545-003, 1:1,000). The cells were washed and mounted with DAPI Fluoromount-G (Southern Biotech).

**Flow cytometry**

Mouse microglia were collected after treatment and washed with PBS. Next, cells were fixed, permeabilized (Invitrogen, Intracellular Fixation & Permeabilization Buffer Set), and stained with intracellular antibodies, followed by overnight incubation at 4 °C in the dark. The following antibodies were used: anti-CD16/32-PerCPCy5.5 (Biolegend 101324, clone: 93, 1:200), anti- Foxp3-BV421 (Biolegend 126419, clone: MF14, 1:200), anti-CD206-PECy7 (Biolegend 141720, clone: CO68C2, 1:200), anti-IL-17A-APCCy7 (Biolegend 506940, clone: TC11-18, 1:200), anti-TREM2-PE (R&D Systems FAB17291P, 1:200), anti-IFNγ-APC (Biolegend 505809, clone: XMG1.2, 1:200), anti-IL-10-APC (Biolegend 505010, clone: JES5-16E3), anti-STAT6-PE (BD Biosciences 612701, clone: pY641, 1:200), anti-ARG1-PerCPCy5.5 (eBioscience 46-3697-82, clone: AlexF5, 1:200), anti-CD103-PE-Cy7 (Biolegend 121425, clone: 2E7, 1:200), and anti-STAT1-PE-CF594 (BD Biosciences 562674, clone: pY701, 1:200). Fluorochrome compensation was performed using single-stained OneComp eBeads (Thermo Fisher Scientific). Data analysis was performed using the FlowJo software (version 10.0).

**Western blotting**

Proteins were extracted from microglial cultures after treatment with RIPA lysis buffer (Sigma) containing a protease inhibitor. A total amount of 40 µg protein from each sample was used for the western blotting experiments. Western blotting was performed using standard sodium dodecyl sulfate-polyacrylamide gel electrophoresis and enhanced chemiluminescence detection reagents (Invitrogen). In brief, polyvinylidene difluoride membranes were blocked in 5% bovine serum albumin for 1 h at room temperature and then incubated at 4 °C overnight with primary antibodies. Next, the membranes were incubated with anti-rabbit, -mouse, or -sheep secondary antibodies conjugated with horseradish peroxidase for 1 h at room temperature (1:5,000, Proteintech). After three washes in TBST, the protein bands were scanned using a chemiluminescence imaging system 9201-550U (G-BOX Chemi XX6/XX9, Syngene, Bangalore, India).The primary antibodies used in this study were: rabbit anti-STAT1 (phospho Tyr701; Cell Signaling Technology 9167, 1:1,000), rabbit anti-STAT1 (Cell Signaling Technology 9172, 1:1,000), rabbit anti-STAT3 (phospho Tyr705; Cell Signaling Technology 9145, 1:1,000), rabbit anti-STAT3 (Cell Signaling Technology 4904, 1:1,000), rabbit anti-STAT6 (phospho Tyr641) (Cell Signaling Technology 9361, 1:1,000), rabbit anti-STAT6 (Cell Signaling Technology 5397, 1:1,000), sheep anti-TREM2 (R&D Systems AF1729, 1:1,000), rabbit anti-P65 (phospho Ser536; Cell Signaling Technology 3033, 1:1,000), rabbit anti-P65 (Cell Signaling Technology 8242, 1:1,000), rabbit anti-P16 (Abcam ab211542, 1:1,000), rabbit anti-βgal (Proteintech 15518-1-AP, 1:1,000), and mouse anti-GAPDH (Proteintech 60004-1-Ig, 1:10,000). Immunoreactivity was assessed using the ImageJ software.

**RT-PCR**

Total RNA was isolated from primary cultured microglia after treatment using an RNA Quick Purification kit (ES science RN001) according to the manufacturer’s instructions. A total of 1 μg RNA (OD260 nm/280 nm = 1.8-2.2) was applied to synthesize the first strand cDNA in a 20 µL system using the Fast Reverse Transcription kit (ES science RT001). The program for reverse transcription was set at 42 °C for 15 min, 85 °C for 10 s, and 4 °C for maintenance. RT-PCR was performed on a QuantStudio 5 (ABI) quantitative PCR machine using SYBR green PCR Master Mix (with ROX) (Dongsheng Biotech P2102) with 0.3 μL of the synthesized cDNA in each reaction. The following program was used: 95 °C for 30 s, 95 °C for 5 s, and 60 °C for 30 s, repeated for 40 cycles; 95 °C for 15 s, 60 °C for 30 s, and 95 °C for 15 s (melt curve). The primers used in this study are listed in Table S7. The cycle time (CT) values were normalized to those of *GAPDH* in the same sample as an internal control, and the values of delta CT log2 normalized to the means of the control group were further subjected to log2 transformation and displayed as a heatmap.

**ELISA**

The supernatants of cultured microglia after treatment were collected by centrifugation (3,000 rpm, 10 min) and stored at -80 °C before examination of TXA2 concentration. A Mouse TXA2 ELISA Kit (HAKATA HZ-030662) was used, and ELISA was performed according to the manufacturer's instructions.

**Statistical analysis**

Statistical tests were performed in R 3.3.2 using R Studio 1.0.136 and GraphPad Prism 8.0. The Wilcoxon rank-sum test was used for comparisons between two groups, while one-way ANOVA (using nonparametric tests) was used for comparisons among three or more groups. Spearman’s correlations between discriminant fecal metabolites and bacteria were calculated using GraphPad Prism 8.0, and the clustering correlation heatmap with signs was constructed using the OmicStudio tools at <https://www.omicstudio.cn>. ROC analysis was conducted to evaluate the differentiated performance of fecal metabolites and bacteria, using OmicStudio tools (<https://www.omicstudio.cn>). Venn diagrams, line regression plots, and distribution trends of the metabolites and bacteria on the age trajectory were plotted using Hiplot (https://hiplot.com.cn/basic/gene-trend). For all analyses, a P value <0.05 was considered statistically significant.
